# Tissue tropisms opt for transmissible reassortants during avian and swine influenza A virus co-infection in swine

**DOI:** 10.1371/journal.ppat.1007417

**Published:** 2018-12-03

**Authors:** Xiaojian Zhang, Hailiang Sun, Fred L. Cunningham, Lei Li, Katie Hanson-Dorr, Matthew W. Hopken, Jim Cooley, Li-Ping Long, John A. Baroch, Tao Li, Brandon S. Schmit, Xiaoxu Lin, Alicia K. Olivier, Richard G. Jarman, Thomas J. DeLiberto, Xiu-Feng Wan

**Affiliations:** 1 Department of Basic Sciences, College of Veterinary Medicine, Mississippi State University, Starkville, Mississippi State, Mississippi, United States of America; 2 College of Veterinary Medicine, South China Agricultural University, Guangzhou, Guangdong, China; 3 Mississippi Field Station, National Wildlife Research Center, Wildlife Services, Animal and Plant Health Inspection Service, United States Department of Agriculture, Starkville, Mississippi State, Mississippi, United States of America; 4 National Wildlife Research Center, Wildlife Services, Animal and Plant Health Inspection Service, United States Department of Agriculture, Fort Collins, Colorado, United States of America; 5 Department of Microbiology, Immunology, and Pathology, Colorado State University, Fort Collins, Colorado State, Colorado, United States of America; 6 Department of Pathobiology and Population Medicine, College of Veterinary Medicine, Mississippi State University, Starkville, Mississippi State, Mississippi, United States of America; 7 Viral Diseases Branch, Walter Reed Army Institute of Research, Silver Spring, Maryland, United States of America; Emory University School of Medicine, UNITED STATES

## Abstract

Genetic reassortment between influenza A viruses (IAVs) facilitate emergence of pandemic strains, and swine are proposed as a “mixing vessel” for generating reassortants of avian and mammalian IAVs that could be of risk to mammals, including humans. However, how a transmissible reassortant emerges in swine are not well understood. Genomic analyses of 571 isolates recovered from nasal wash samples and respiratory tract tissues of a group of co-housed pigs (influenza-seronegative, avian H1N1 IAV–infected, and swine H3N2 IAV–infected pigs) identified 30 distinct genotypes of reassortants. Viruses recovered from lower respiratory tract tissues had the largest genomic diversity, and those recovered from turbinates and nasal wash fluids had the least. Reassortants from lower respiratory tracts had the largest variations in growth kinetics in respiratory tract epithelial cells, and the cold temperature in swine nasal cells seemed to select the type of reassortant viruses shed by the pigs. One reassortant in nasal wash samples was consistently identified in upper, middle, and lower respiratory tract tissues, and it was confirmed to be transmitted efficiently between pigs. Study findings suggest that, during mixed infections of avian and swine IAVs, genetic reassortments are likely to occur in the lower respiratory track, and tissue tropism is an important factor selecting for a transmissible reassortant.

## Author summary

Genetic reassortments between avian and swine influenza viruses are likely to occur in the swine lower respiratory track, and tissue tropism is an important factor selecting for a transmissible reassortant; determination of tissue tropisms for potential reassortants between contemporary avian and swine influenza viruses would help identify transmissible reassortants with public health risks.

## Introduction

Influenza A viruses (IAVs) are negative-strand RNA viruses with eight gene segments of various lengths, ranging from about 890 to 2,341 nucleotides. IAV subtypes are determined by the virus surface glycoproteins, hemagglutinin (HA), and neuraminidase (NA). A total of 18 HA (H1–H18) and 11 NA (N1–N11) subtypes have been identified [1, 2]. IAVs with subtypes H1–H16 and N1–N9 have been recovered from a variety of bird species, including at least 105 wild bird species of 26 different families [3]. Among wild birds, those living in wetlands and aquatic environments (such as the *Anseriformes*, particularly ducks, geese, and swans; and *Charadriiformes*, particularly gulls, terns, and waders) constitute the major natural IAV reservoir [4].

In addition to birds, IAVs can also cause infections in marine mammals, land-based lower mammals (e.g., pigs, dogs, and horses), and humans. Wild birds maintain a large IAV genetic pool, which contributes to the appearance of new IAVs in humans, lower mammals, and domestic poultry. Because pigs have both avian-like receptors (α 2,3-linked sialic acid, SA2,3Gal) and human-like receptors (α 2,6-linked sialic acid, SA2,6Gal) on the epithelial cells of their respiratory tissues, they have been proposed to serve as an intermediate host “mixing vessel” for generating pandemic IAV strains through genetic reassortment between avian and swine or human IAVs. Three of four documented pandemic strains are reassortants between avian and swine or human IAVs: the 1957 subtype H2N2 pandemic IAV probably emerged from H2 avian IAV and H1N1 human IAV [5]; the 1968 subtype H3N2 pandemic IAV probably emerged from H3 avian IAV and the H2N2 human IAV [5]; and the 2009 subtype H1N1 pandemic IAV had PB2 and PA genes from avian IAVs, PB1 gene from human H3N2 IAV, and other genes from swine IAVs [6]. However, emergence of these pandemic viruses is complicated and could require multiple reassortment events (in addition to mutations) across years.

In addition to virus receptor binding attributes, compatibility between the co-circulating avian and swine or human IAVs will determine whether a new reassortant can be generated in swine. A number of studies have been performed to evaluate the compatibility of emerging avian IAVs and endemic human IAVs, such as avian H9N2 versus human H1N1 [7], avian H5N1 versus human H3N2 [8–10], and avian H5N1 versus human H1N1 [11]. The studies demonstrated that gene compatibility between viruses is subtype- and strain-dependent [7, 10–12]; however, transmissible viruses are possibly derived from genetic reassortments between avian and human IAVs [13]. In addition, given two IAVs, the timing and dose of inoculation, as well as inoculation location, can affect the dynamics of IAV reassortment and the reassortants to be generated [14–18]. Thus, genetic reassortment is a complicated biological process, and how a transmissible reassortant emerges in swine are not well understood. Efforts were carried out to reproduce the 2009 H1N1 pandemic strains by mixing two swine IAVs in domestic pigs with or without preexisting immunity, but they were unsuccessful [19].

We conducted this study to test the hypothesis that tissue tropism plays an important role in selecting transmissible reassortants from various reassortants generated in pigs co-infected with avian and swine IAVs. We determined the genotypes and phenotypes of reassortants recovered from a mixed group of pigs (influenza-seronegative, avian H1N1 IAV–infected, and swine H3N2 IAV–infected pigs) and then characterized the temporal and spatial distributions of the reassortants. Feral swine were used as the animal model in this study, and we will simply describe feral swine as pigs or swine in the following sections.

## Results

### Selection of avian and swine IAVs in the animal experiment

**Fig 1:**
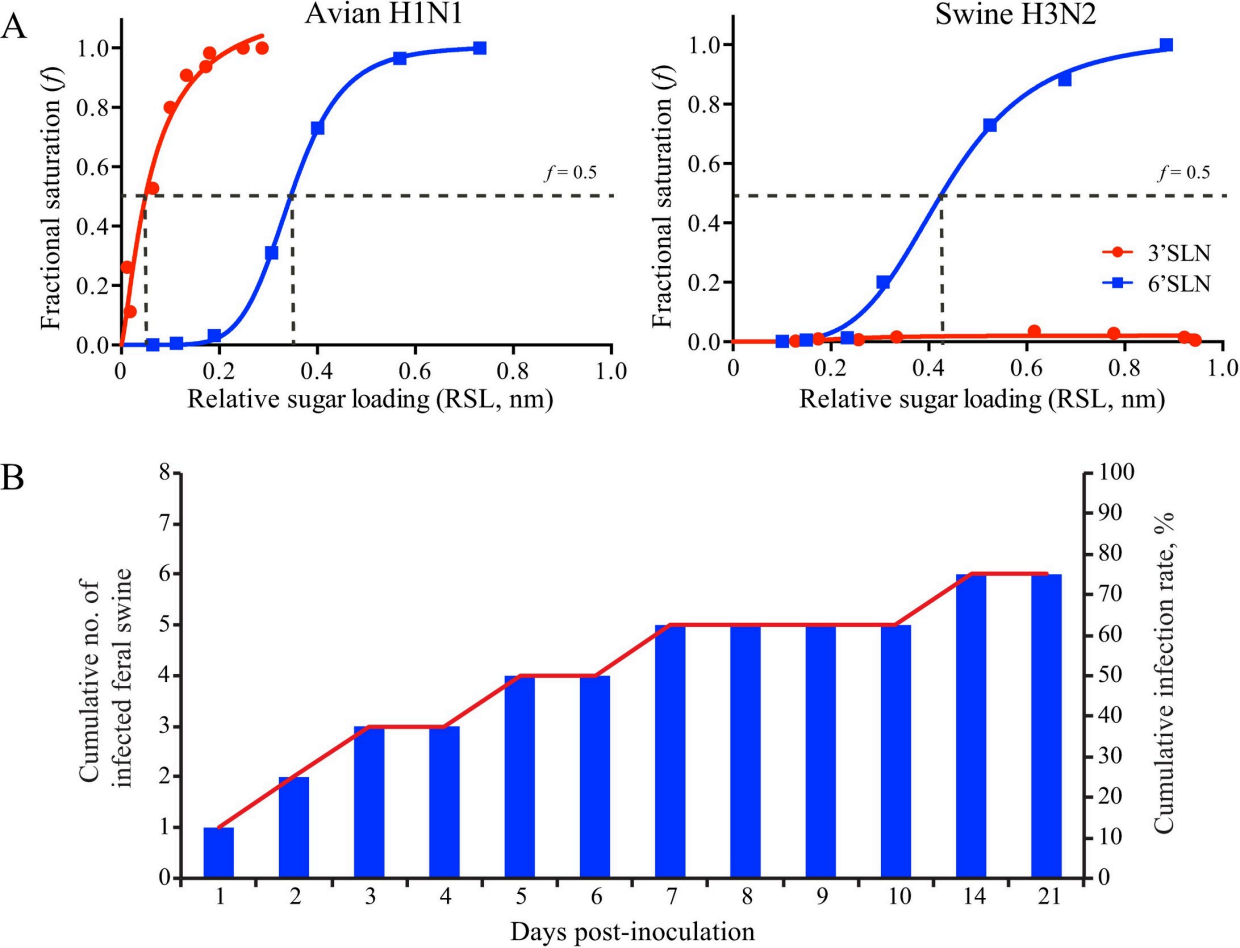
Biological characteristics of avian H1N1 and swine H3N2 influenza A viruses (IAVs). (A) Receptor binding properties of avian H1N1 (left panel) and swine H3N2 (right panel) IAVs. Biolayer interferometry data for the binding of avian H1N1 and swine H3N2 IAVs to avian (3ʹSLN) and human (6ʹSLN) receptor analogues. Streptavidin-coated biosensors were immobilized with biotinylated glycans at different levels. Sugar loading–dependent binding signals were captured in the association step and normalized to the same background. Binding curves were fitted by using the binding-saturation method in GraphPad Prism version 7 (https://www.graphpad.com/scientific-software/prism/). Horizontal dashed line indicates half of the fractional saturation (*f* = 0.5), and vertical dashed line indicates relative sugar loading (RSL_0.5_) at *f* = 0.5; the higher the RSL_0.5,_ the smaller the binding affinity. (B) Infectivity of avian H1N1 IAV in feral swine. The animals in treatment group were intranasally inoculated with 10^6^ EID_50_ of avian H1N1 IAV, and animals in the control group were intranasally inoculated with 1 mL of PBS. On indicated days, nasal wash fluids and respiratory tract tissues were collected from each animal for viral titration in 10-day-old specific pathogen–free eggs. Cumulative infectivity calculations were based on titration results.

Infectivity of avian IAVs in swine is strain dependent [20], and avian IAVs to be used in animal studies must show optimum infectivity in swine. To select an avian-origin IAV for animal experiments, we characterized the receptor binding profiles of avian IAVs and selected an avian IAV binding to both avian and human-like receptors. Biolayer interferometry analyses suggested that influenza strain A/mallard/Wisconsin/A00751454/2009(H1N1) had detectable binding affinities to 3ʹ-sialyl-*N*-acetyllactosamine (3ʹSLN, representing avian-like receptor SA2,3Gal) and to 6ʹ-sialyl-*N*-acetyllactosamine (6ʹSLN, representing human-like receptor SA2,6Gal). To further determine whether an IAV prefers more to 3ʹSLN rather than 6ʹSLN (or more to 6ʹSLN rather than 3ʹSLN), we quantified and compared 50% relative sugar loading (RSL) concentration (RSL_0.5_) at half of the fractional saturation (*f* = 0.5) of the testing virus against both 6ʹSLN and 3ʹSLN. The higher the RSL_0.5_, the smaller the binding affinity. Results from quantitative analyses suggested that this avian H1N1 IAV had a higher relative sugar loading (RSL) concentration to 6ʹSLN (RSL_0.5_ = 0.35 nm) than that to 3ʹSLN (RSL_0.5_ = 0.06 nm) (Fig 1A, left panel), indicating that this avian H1N1 IAV has a higher binding affinity to 3ʹSLN than that to 6ʹSLN. We further evaluated the infectivity of A/mallard/Wisconsin/A00751454/2009(H1N1) virus in swine. By combining viral shedding (S1 Table), viral loads in tissues (S1 Fig), serologic analyses data (S2 Table), we showed that A/mallard/Wisconsin/A00751454/2009(H1N1) virus successfully infected 6 of 8 treatment pigs (Fig 1B; S1 Text).

In addition to selecting the avian-origin A/mallard/Wisconsin/A00751454/2009(H1N1) influenza strain for use in the animal study, we also selected a feral swine–origin IAV, A/swine/Texas/A01104013/2012(H3N2) [21]. Biolayer interferometry analyses suggested, in contrast to findings for A/mallard/Wisconsin/A00751454/2009(H1N1), that this swine H3N2 IAV binds to 6ʹSLN but not 3ʹSLN (Fig 1A, right panel). According to earlier findings [21], this swine H3N2 IAV is highly infectious and transmissible in feral swine.

### Animal experiments with avian and swine IAVs in feral swine

To mimic the natural scenario for IAV co-infections, we included one pig inoculated with A/mallard/Wisconsin/A00751454/2009(H1N1), one pig inoculated with A/swine/Texas/A01104013/2012(H3N2), and one or two IAV-seronegative contact pigs in each of five experimental groups. Temporal and spatial dynamics of virus infections and genetic reassortments in these pigs were characterized to understand how a transmissible reassortant was generated between the avian and swine IAVs tested.

Although the initial viral shedding time for individual pigs varied, contact pigs and pigs inoculated with swine H3N2 virus or avian H1N1 virus shed viruses as early as 3 days post-inoculation (dpi) and continued to shed viruses on 7 dpi before all pigs were euthanized (S3 Table). Early viral shedding loads in avian H1N1 virus–inoculated pigs were much lower than those in swine H3N2 virus–inoculated pigs, but by 7 dpi, levels were similar for the two groups (range, 4.032–6.699 log_10_TCID_50_/mL). For individual pigs, virus loads in nasal wash fluids differed widely from those in respiratory tract tissues. However, virus was consistently detected in the tissues of pigs that had positive virus titration results for nasal wash fluids. Furthermore, the pattern of viral loads in tissues were similar to those in nasal wash fluids. No significant clinical signs of infection (e.g., temperature, coughing, and body weight changes) were observed in any pigs in the animal experiment (S2 Fig).

### Temporal and spatial dynamics of genetic reassortments during IAV co-infections

**Fig 2:**
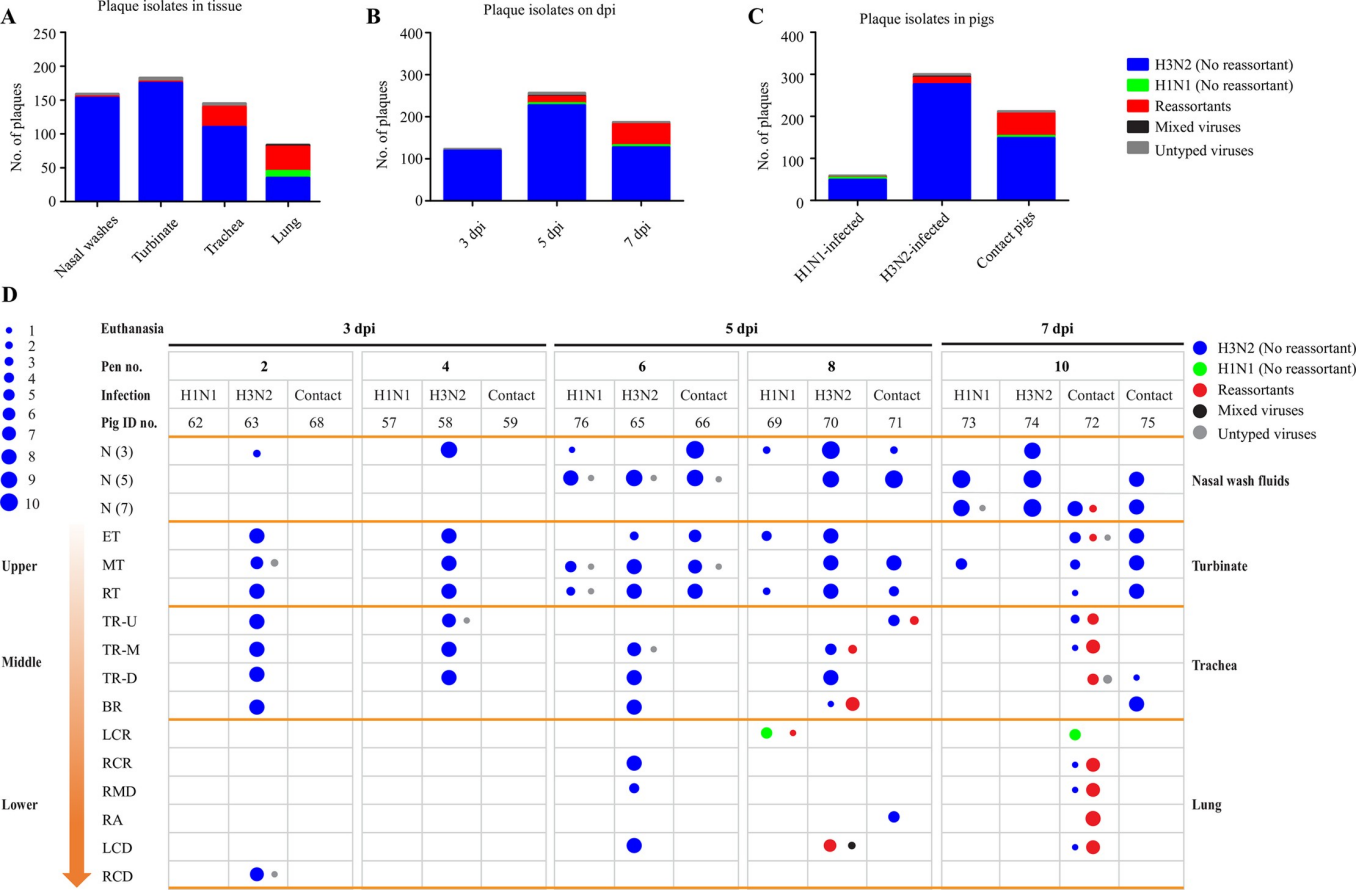
Genotyping of plaque isolates in animal study. A total of 571 viruses from nasal wash specimens (n = 159) and respiratory tract tissues (n = 412) were isolated by plaque assays and then subjected to genomic sequencing. (A) Plaque isolates from tissues of infected pigs. (B) Plaque isolates from infected pigs euthanized on each indicated day. (C) Plaque isolates from contact pigs, H1N1-inoculated pigs, and H3N2-inoculated pigs. (D) Overall genotyping. Grouping information is shown at the top of the figure. Nasal wash fluids (N) obtained 3, 5, and 7 days after inoculation (dpi) and respiratory tract tissue sections are indicated on the left. Dot size indicates quantity of isolates; colors indicate different origins of the isolates. ET, ethmoid turbinate; MT, middle turbinate; RT, rostral turbinate; TR-U, upper trachea; TR-M, middle trachea; TR-D, distal trachea; BR, bronchus; LCR, left cranial lung; RCR, right cranial lung; RMD, right middle lung; RA, right accessory; LCD, left caudal lung; RCD, right caudal lung.

To understand the bottlenecks for viral reassortment between avian and swine IAVs and to elucidate how transmissible IAVs emerge from reassortment events in swine, we characterized the temporal and spatial dynamics of genetic reassortments during the IAV co-infections. Specifically, individual virions from samples with positive viral titration results were purified using plaque assays. To minimize possible sampling biases, we collected a maximum of 10 plaques for each individual nasal wash fluids or tissue sample. In total, 571 plaques were generated: 59 from avian H1N1 virus–inoculated pigs, 300 from swine H3N2 virus–inoculated pigs, and 212 from contact pigs. Of the 571 plaques, 157 were recovered from samples collected on 3 dpi, 264 from samples collected on 5 dpi, and 150 from samples collected on 7 dpi; these plaques were broadly distributed among the nasal wash fluids (n = 159) and the upper (i.e. turbinate) (n = 188), middle (i.e. trachea) (n = 137), and lower (i.e. bronchus, left cranial lung, right cranial lung, right middle lung, right accessory, left caudal lung, and right caudal lung) (n = 87) respiratory tracts of the pigs (Fig 2; S1 Text).

**Fig 3:**
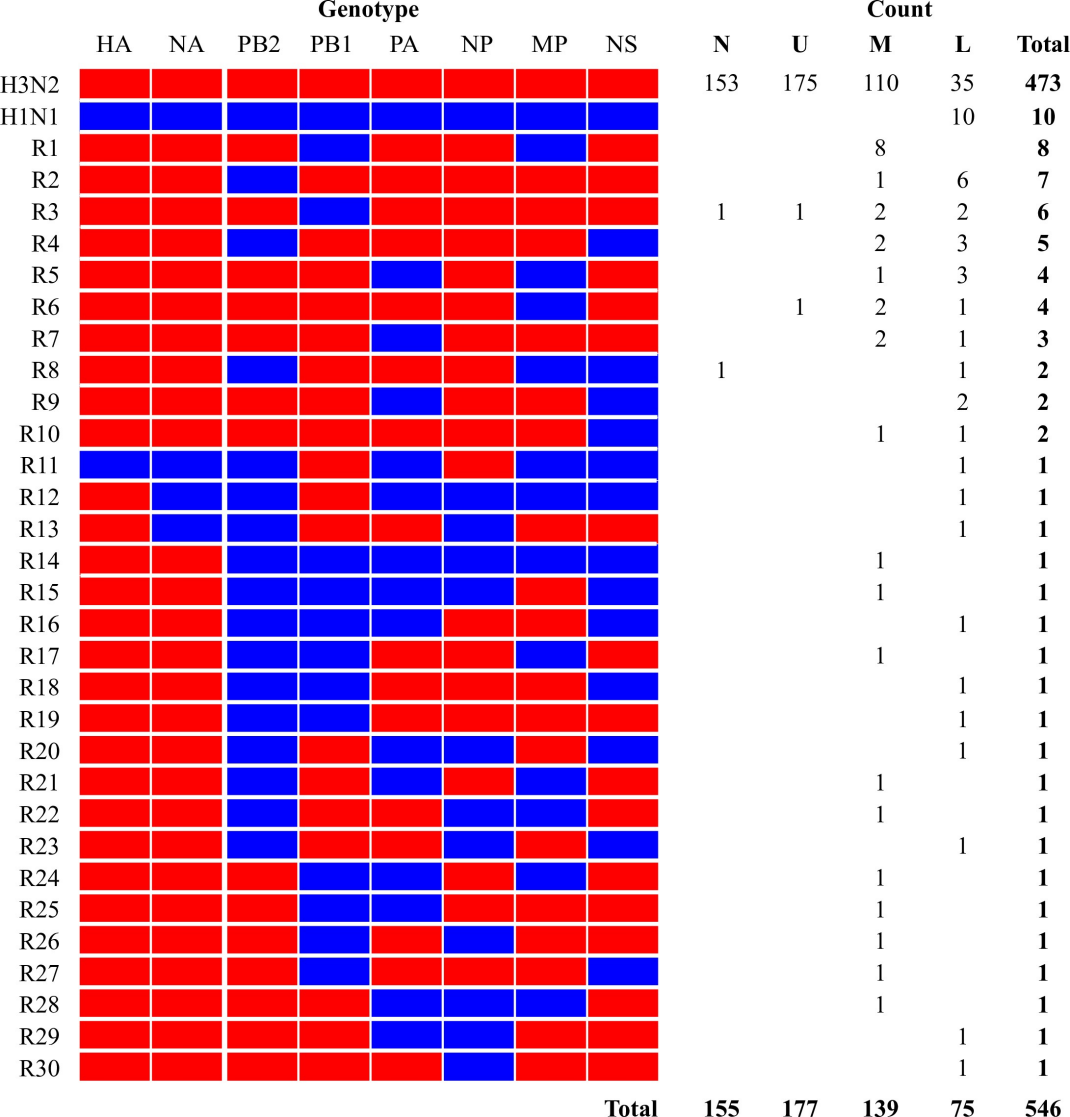
Genotypes of isolated viruses. A total of 571 plaques were analyzed and 546 of them were genotyped with complete genomic sequences. The origin of each of the eight gene segments is shown at the top of the columns on the left. Red indicates segments from swine H3N2 virus, blue indicates segments from avian H1N1 virus. The numbers in the columns right indicate the quantity of isolates in nasal wash fluids (N) and in the upper (U, turbinate), middle (M, trachea), and lower (L, lung) respiratory tracts of pigs. Each plaque was assigned with a plaque number and a genotype number, and one genotype can have multiple plaques. R-number on the left column indicates a specific reassortant genotype number. For example, R3 refers to genotype 3 and includes a total of six individual plaques.

The 571 viruses were subjected to whole-genomic sequencing, and sequences were obtained for 546 viruses with complete genomic sequences. The stock of the H1N1 inoculum and the H3N2 inoculum were also subjected to whole-genomic sequencing, the data from which were used in genotype analyses and polymorphism characterization for all of the 546 plaque isolates. The viruses were distributed among 32 different genotypes, one of which was the same as that for the parent avian H1N1 virus and included 10 (1.83%) of the 546 viruses. Another genotype was the same as that for the parent swine H3N2 virus and included 473 (86.63%) of the 546 viruses. The other 30 genotypes were novel, with at least one gene from each of the two parent viruses, and included 63 (11.54%) of the 546 viruses. Of the 63 reassortant viruses, 60 contained HA and NA genes from swine H3N2 viruses; one contained HA and NA from avian H1N1 virus; two contained HA from swine H3N2 virus but NA from avian H1N1 virus. Each plaque was assigned with a plaque number and a genotype number, and one genotype can have multiple plaques. For example, R3 refers to genotype 3 annotated in Fig 3 and includes a total of six plaques (plaque #39, #43, #69, #258, #456, and #551; S9 Table).

The 473 parental swine H3N2–like viruses (86.63% of the 546 sequenced viruses) were identified not only in pigs inoculated with swine H3N2 virus but also in both pigs inoculated with avian H1N1 virus and the contact pigs; these viruses were identified in nasal wash samples and in upper, middle, and lower respiratory tract tissue samples. Avian parental H1N1–like viruses were identified not only on 5 dpi in the lung of the pig inoculated with avian H1N1 virus but also in the lung of one contact pig euthanized on 7 dpi. Of interest, of the 63 reassortant viruses, 11 were recovered from one H3N2 virus–inoculated pig euthanized on 5 dpi, one was from an H1N1 virus–infected pig euthanized on 5 dpi, three were from a contact pig euthanized on 5 dpi, and 48 were from a contact pig euthanized on 7 dpi.

The viruses recovered from the lower respiratory tract had higher genomic diversity (19 genotypes) than those recovered from the middle respiratory tract (18 genotypes). The least genomic diversity (i.e. only two reassortants) were identified in each of the nasal wash samples and upper respiratory tract samples. At least eight genotypes of reassortants were identified in samples from two or more anatomical locations: nasal wash fluids or upper, middle, or lower respiratory tract tissues. Of note, one genotype of reassortants (genotype R3) was identified in nasal wash fluids and in all upper, middle, and lower respiratory tract tissues (Fig 3).

### Replication kinetics of reassortant viruses *in vitro*

**Fig 4:**
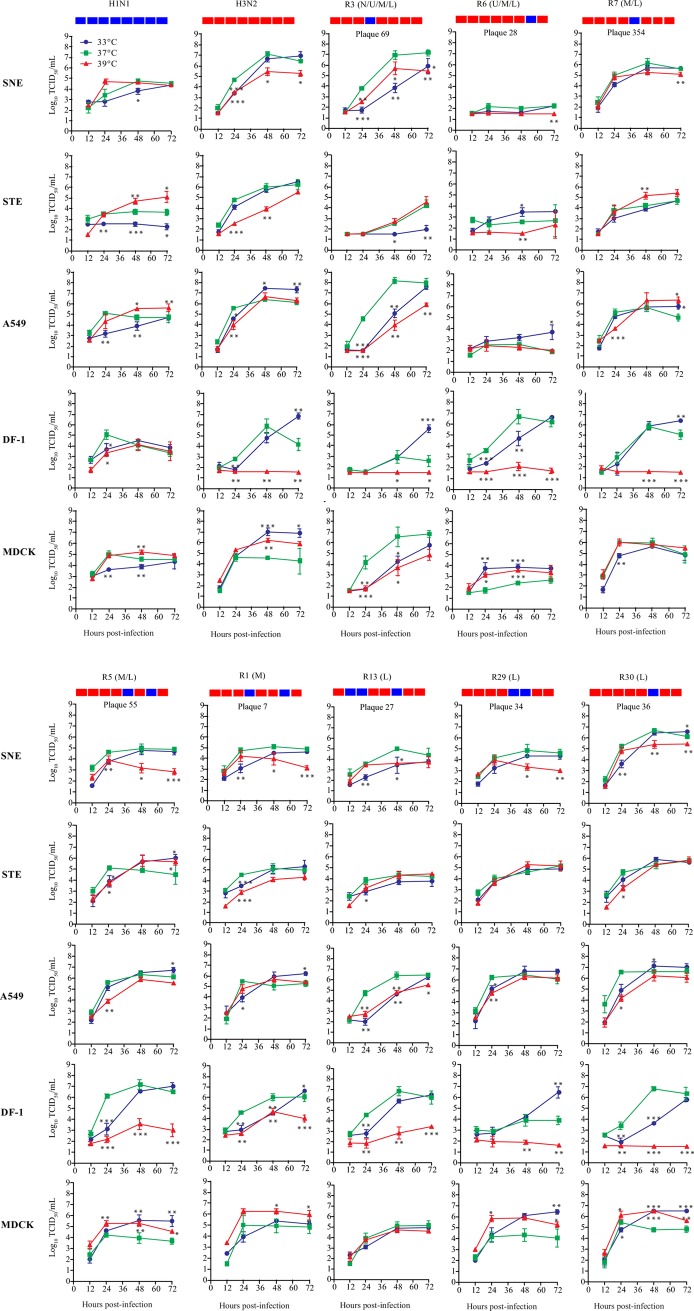
Growth dynamics of parental and reassortant viruses in swine nasal epithelium cells (SNE), swine tracheal epithelium cells (STE), human alveolar basal epithelial cells (A549), chicken embryo fibroblasts (DF-1), and Madin-Darby canine kidney (MDCK) cells. Cells were infected at a multiplicity of infection of 0.001 TCID_50_/cell with the indicated parental or reassortant viruses. The nasal wash fluids (N) and tissue sections of the testing genotypes of the reassortant viruses are shown (U, upper respiratory tract; M, middle respiratory tract; L, lower respiratory tract). The origin of each segment for indicated viruses are shown at the top of the columns; red indicates segments from swine H3N2 virus, and blue indicates segments from avian H1N1 virus. Infected cells were incubated at 33°C, 37°C, or 39°C. Growth curves were determined by using the viral titers in the supernatants of infected cells obtained at 12, 24, 48, and 72 h post-inoculation. Data shown represent the mean titers ± standard errors (n = 3 cultures). Significance is noted (*P<0.05, **P<0.01, and ***P<0.001) where virus titers obtained for a virus at 33°C or 39°C were statistically different from those obtained at 37°C at the same time point.

**Fig 5:**
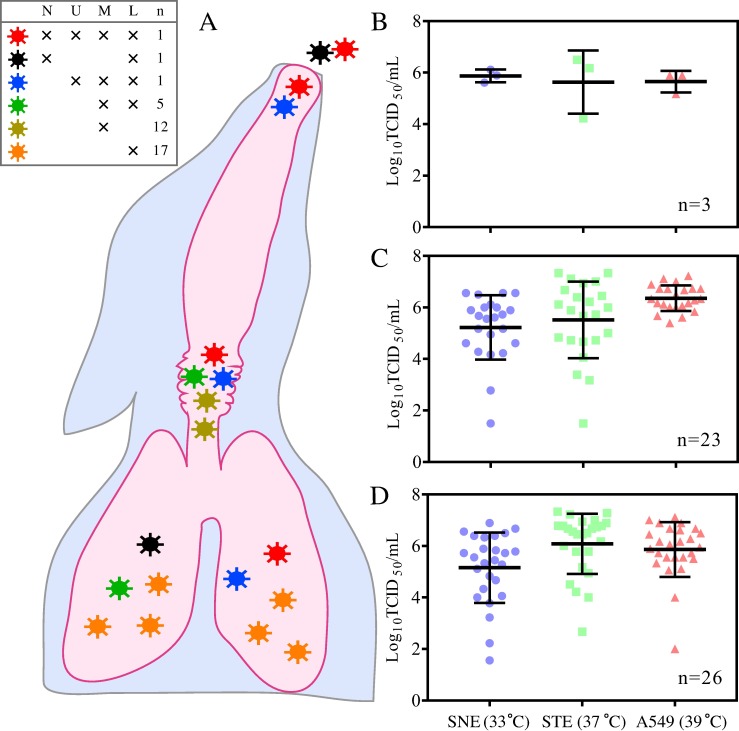
Growth dynamics of reassortant influenza viruses in swine nasal epithelium cells (SNE), swine tracheal epithelium cells (STE), and human alveolar basal epithelial cells (A549). A) Plaque isolates from nasal wash fluids (N) and upper (U), middle (M), and lower (L) respiratory tracts of infected feral swine were selected to grow in SNE, STE, and A549 cells at 33°C, 37°C, and 39°C, respectively. Stars indicate viruses isolated from various samples. Colors indicated virus genotypes. No. of genotypes were also shown in the legend. B–D) Virus titers for reassortants from nasal wash fluids and upper (B), middle (C), and lower (D) respiratory tracts. Cells were seeded in 6-well plates and infected in triplicate with plaque viruses at a multiplicity of infection of 0.001. Supernatant samples were collected at 72 h and titrated by TCID_50_ in Madin-Darby canine kidney cells. In panel B-D, the color was used to distinguish the factor of temperature (i.e., blue for 33 ^o^C, green for 37 ^o^C, and red for 39 ^o^C) and the shape to distinguish the tissue source for the plaques (i.e., circle for upper respiratory tracts, square for middle respiratory tracts, and triangle for lower respiratory tracts). The distribution of the plaques are shown in Fig 2, the genotypes of the plaques in Fig 3, and the growth phenotypes of the plaques in Fig 4 and S3 Fig.

To test the working hypothesis that tissue tropism presents a bottleneck for genetic reassortment and selects the viruses to be shed, we performed viral growth analyses for parent avian H1N1 and swine H3N2 wild-type influenza viruses and for 52 representative reassortant viruses in swine nasal epithelium (SNE) cells at 33°C, swine tracheal epithelium (STE) cells at 37°C, and human alveolar basal epithelial (A549) cells at 39°C. Because of a lack of swine alveolar epithelial cells, human A549 cells were used to mimic swine alveolar epithelial cells from swine lower respiratory tracks. Human A549 cells are characterized as a type II pulmonary epithelial cell model study on IAV infection [22], and both human and swine type II pneumocytes expressed α-2,3- and α-2,6-linked sialic acid receptors [23, 24], and are targets of IAV infection [23, 25–27]. Three incubation temperatures were applied to simulate the temperatures of airway in pigs: approximately 33°C in the nasal, 37°C in the trachea, and 39°C in the lung. Peak virus titers in the supernatants were determined at 72 h post-inoculation using TCID_50_ in Madin-Darby canine kidney (MDCK) cells (Figs 4 and 5; S3 Fig).

Of the 52 testing reassortants, 51 showed efficient replication in A549 cells at 39°C, with virus titers ranging from 4.0 to 7.0 log_10_TCID_50_/mL (S3 Fig); plaque #28 (genotype R6, Fig 4) was the one exception. The average virus titer for viruses from the upper respiratory track was 5.65 (± standard deviation; ±0.41) log_10_TCID_50_/mL. Average titers for viruses from the middle and lower respiratory tracks were 6.36 (±0.57) log_10_TCID_50_/mL and 6.62 (±0.76) log_10_TCID_50_/mL, respectively.

However, the same testing reassortant viruses showed relatively large variations in infectivity in STE cells at 37°C (virus titer range, 1.5–7.5 log_10_TCID_50_/mL) (S3 Fig). The average virus titer for viruses from the upper respiratory track was 5.63 (±1.14) log_10_TCID_50_/mL, and average titers for viruses from the middle and lower respiratory tracks were 5.52 (±1.70) log_10_TCID_50_/mL and 6.22 (±1.00) log_10_TCID_50_/mL, respectively.

All viruses recovered from the upper respiratory tract samples replicated well in SNE cells at 33°C (average virus titer [±standard deviation], 5.89 [±0.49] log_10_TCID_50_/mL), but viruses from the middle and lower respiratory tracts showed large variations in infectivity (virus titer range, 1.5–7.0 log_10_TCID_50_/mL) (S3 Fig). The average virus titer for viruses from middle respiratory tracts was 5.22 (±1.31) log_10_TCID_50_/mL, and that for viruses from lower respiratory tracts was 5.27 (±1.29) log_10_TCID_50_/mL. Of note, the testing reassortants recovered from the upper respiratory tract samples replicated well in all three temperature conditions (virus titer range, 4.0–7.0 log_10_TCID_50_/mL).

### Transmission of shed reassortant virus in feral swine

We hypothesized that the nasally shed reassortant viruses would be highly transmissible among feral swine. To test this hypothesis, we evaluated the transmission ability of reassortant plaque #69 (genotype R3) among feral swine. Eight feral swine were separated into four experiment groups comprised of two pigs each: one plaque #69 (genotype R3) virus–inoculated pig and one IAV-seronegative pig as the contact pig. Four additional feral swine were used as non-experiment group control pigs and housed in a different facility. In the four experiment groups, viral shedding was initially detected in nasal wash samples from plaque #69 (genotype R3) virus–inoculated pigs on 3 dpi (virus titer range, 2.67–4.67 log_10_TCID_50_/mL), and from the contact pigs on 5 dpi (virus titer range, 2.5–6.0 log_10_TCID_50_/mL). The virus-inoculated pigs continued to shed viruses until 5 dpi, and the contact pigs shed viruses until 9 dpi (S6 Table). Two plaque #69 (genotype R3) virus–inoculated pigs were euthanized on 3 and 5 dpi. Results from viral titration showed that only the upper respiratory tract tissues from the pig euthanized on 3 dpi were IAV-positive by virus titration (5.0 log_10_TCID_50_/mL) and, however, that all the upper, middle, and lower respiratory tract tissues from the pig euthanized on 5 dpi were IAV-positive (titer range, 3.5–5.0 log_10_TCID_50_/mL) (S7 Table). The remaining four pigs in the experiment groups seroconverted on 21 dpi (HI titer range, 1:640–1:1,280) (S5 Fig and S8 Table). No viruses were detected in nasal wash fluids or tissue samples from the four non-experiment group control pigs, and they all remained seronegative for plaque #69 (genotype R3) virus 21days after inoculation of pigs in the experiment group. According to viral titration and seroconversion results, all plaque #69 (genotype R3) virus-inoculated pigs became virus infection positive on 3 dpi (S5A Fig), while all contact pigs became virus infection positive on 5 dpi (S5B Fig). In summary, these results showed that the testing reassortant virus could be transmitted directly between pigs with an efficiency of 100%.

## Discussion

Swine have been proposed as a “mixing vessel,” generating reassortants between avian IAVs and swine or human IAVs. Such reassortant viruses caused the 2009 pandemic outbreaks among humans [6]. The detection of avian IAVs in swine is not uncommon, and subtype H2N3 [28], H4N6 [29], H1N1 [30, 31], H1N2 [32], H3N3 [33], H5N1 [34], H6N6 [35], and H9N2 [36, 37] IAVs have been reported in domestic swine. Under laboratory conditions, avian IAV subtypes H1–H13 can infect and replicate in swine at different levels of susceptibility [20]. However, among these avian IAVs, only subtypes H1N1 and H3N2 have become enzootic in domestic swine; the other avian-origin IAVs have transiently infected domestic swine. Novel reassortants with avian IAVs genes have rarely been detected in swine. By housing IAV-seronegative pigs together with H3N2 IAV–inoculated and avian H1N1 IAV–inoculated pigs, we demonstrated that 1) a large number of reassortants were generated in the pigs’ lower respiratory tracts, but only a few of the reassortants were shed; and 2) tissue tropisms, especially replication ability in swine nasal epithelium cells at 33°C, are keys to selecting a reassortant virus that can be shed.

Reassortment between two IAVs of different or the same origins have been well documented [7–9, 11, 14–19, 38]. Previous studies tested the compatibility of emerging avian IAVs and endemic human influenza viruses, such as the compatibility of avian H9N2 with human H1N1 [39, 40], avian H5N1 with human H3N2 [8, 10, 41], and avian H5N1 with human H1N1 [42]. Gene compatibility between viruses has been shown to be subtype- and strain-dependent; however, transmissible viruses are possibly derived from genetic reassortments between avian and mammalian IAVs [9]. Results from our study suggested that 30 of 254 possible reassortant genotypes between an avian H1N1 IAV and a swine H3N2 IAV were detected, and most were in the middle and lower respiratory tracts of the experiment pigs; only two genotypes of reassortants (i.e., genotypes R3 and R8; Fig 3) shed from these pigs, and these genotypes of reassortants were also detected in middle (genotype R3) and/or lower respiratory tracts (genotypes R3 and R8) of the pigs. Study findings suggest that reassortments between avian and swine IAVs are tissue-dependent and non-random, and, in swine, they occur most frequently in the lower respiratory tract.

Both *in vivo* and *in vitro* studies have suggested that the timing and dose of inoculation, as well as inoculation location, contribute to the efficiency of reassortment between influenza viruses [15–18]. In this study, we mimicked the natural scenario of reassortment generation by housing IAV-seronegative pigs with swine H3N2 IAV–infected pigs and avian H1N1 IAV–infected pigs. The IAV reassortant viruses were detected as early as 5 dpi, and more on 7 dpi; reassortants were detected not only in the contact pigs but also in the pigs infected with swine H3N2 IAV and those infected with avian H1N1 IAV. Such results suggest that genetic reassortments can readily occur and generate genomically diverse reassortants if avian IAVs are introduced into a herd with circulating swine IAVs.

In this study, the distribution of reassortants indicate obvious tissue tropism, with most reassortants occurring in middle and lower respiratory tissues and a few in upper respiratory tissue. We conducted further viral growth studies for 52 representative reassortants by using an *in vitro* infection model of swine with three cell lines imitating the upper (i.e. SNE), middle (i.e. STE), and lower (i.e. A549) respiratory tracts of swine. Results indicate that the reassortants detected in middle and lower respiratory tracts showed large variations in growth properties in SNE at 33°C and STE at 37°C; however, the reassortants detected in nasal wash fluids and turbinate can replicate well in SNE at 33°C and in STE at 37°C (Figs 4 and 5). These results suggest that tissue tropism contributes to the unbalanced distribution of reassortants through swine respiratory tract tissues. Receptor binding properties have been reported to be one of the key factors determining IAV host and tissue tropisms [43]. However, most (60 of 63) of the recovered reassortants from this study had HA and NA genes from swine H3N2 IAVs thus having the same receptor binding properties; receptor binding properties of IAVs do not seem to play a key role in the unbalanced distribution of these reassortants in respiratory tracts of feral swine [19, 38]. Instead, it is likely the other gene segments, such as the ribonucleoprotein complex [44] and NS gene [45], could interact with the host genes and formulate such tissue tropisms. Influenza surveillance showed a large number of genetic variants of subtype H1 and H3 IAVs are co-circulating and enzootic in domestic swine in the United States [46, 47]. Phenotype analyses suggested that these reassortants can have large variation in tissue tropisms and transmissibility in mammals [48–50]. Thus, in addition to receptor binding properties, genetic constellation is an important factor determining influenza tissue and host tropisms.

Adapted mutations were also reported to affect viral infectivity and tissue tropisms [44]. A number of mutations or polymorphisms were observed across IAV gene segments (S1 Text). Of interest, in swine H3N2 (no reassortment) isolates, mutation Q197R in HA protein exhibits obvious tissue-dependent distribution: the mutation occurs more frequently in virus recovered from nasal wash fluids and upper respiratory tracts than in those recovered from middle and lower respiratory tracts (S4 Table). However, analyses of genomic sequences of human and swine H3N2 IAVs in the public databases showed that 93.11% of human H3N2 IAVs and 97.95% of swine H3N2 IAVs possess glutamine (Gln, Q) at residue 197 of HA protein, but just 4.86% of human H3N2 IAVs and 0.61% of swine H3N2 IAVs possess arginine (Arg, R) at the same site of HA protein (S5 Table). We further compared the viral growth kinetics of plaque viruses with or without the Q197R mutation on HA protein under three different temperature conditions (S4 Fig). Peak virus titers at 72 h post-inoculation for the viruses with the mutation did not increase growth efficiency; thus, this mutation does not seem to play an important role in determining the tissue tropisms of IAVs.

In the United States, in recent years, it has been challenging to identify influenza seronegative domestic pigs due to influenza enzootics and nationwide implementation of influenza vaccination in domestic pigs. In this study, feral swine was used as the animal model in the animal experiment because it is much easier to obtain influenza seronegative feral swine. However, although we confirmed that these feral swine had not been exposed to brucellosis, pseudorabies and influenza A virus [21], it is likely that these animals had been exposed to other pathogens, especially parasites. Of interests, prior studies suggested that environment changes for the animals used in the experiments affected the experimental results and that non-specific pathogen free animals could have immune systems closer to those of adult humans [51]. Nevertheless, results from our feral swine experiments clearly demonstrated that tissue tropism plays an important role in selecting transmissible reassortants from various reassortants generated in pigs co-infected with avian and swine IAVs.

Compared with domestic pigs, feral swine play an important role in influenza ecology because of their increasing population and ability to roam freely [21, 52]. In one study, 7 (19%) of 37 feral swine had direct contact with domestic swine [53]; however, feral swine have more opportunities to become infected with avian IAVs because they can easily come into contact with feces or water contaminated by IAV-infected wild birds or with infectious dead birds. These characteristics make feral swine a potential intermediate host for reassortment and transmission of avian IAVs or genes to humans.

Virologic and serologic surveillance showed evidence of feral swine exposed to both H3N2 and H1N1 IAVs, predominately H3N2 IAVs [52, 54]. Furthermore, the H3N2 virus recovered from feral swine shared viral shedding pattern and antibody response dynamics similar to those for viruses isolated from domestic pigs [21, 52]. Since 2009, the pandemic H1N1 virus has been identified in domestic [52, 55, 56] and feral swine [57] in the United States. Therefore, avian and swine IAVs are continuously transmitted to feral swine, and this finding highlights the important role of feral swine in influenza ecology. Our findings indicate that reassortants between avian and swine IAVs can be shed nasally and be transmitted between feral swine with 100% efficiency under experimental conditions. Thus, feral swine could serve as a potential intermediate host for generating and transmitting reassortant viruses containing both avian and swine influenza virus genes to domestic swine and, ultimately, to humans.

In summary, our study findings illustrate how transmissible reassortants arise between avian and swine IAVs in pigs. The reassortments occur in lower respiratory tracts, and tissue tropisms could have selected which reassortant viruses would be shed from the pigs. Determination of tissue tropisms for potential reassortants from contemporary avian and swine IAVs would help identify transmissible reassortants with public health risks.

## Materials and Methods

### Ethics statement

The animal experiments were performed under the protocol numbers QA2296 titled Potential of Avian influenza A virus to infect feral swine and QA2494 titled Potential for feral swine and avian H3N2 influenza to cause a mixed infection in feral swine, both of which approved by the Institutional Animal Care and Use Committee of National Wildlife Research Center (NWRC) in accordance with the USDA Animal Welfare Regulations. Biosafety protocol for laboratory and animal experiments: Virus titration and purification and virus inoculation in feral swine were conducted under Biosafety Level 2 conditions, in compliance with U.S. Department of Agriculture–approved protocols of Institutional Animal Care and Use Committee and Institutional Biosafety Committee.

### Cells

Madin-Darby canine kidney (MDCK) cells, human alveolar adenocarcinoma (A549) cells, and chicken embryo fibroblasts (DF-1) cells were obtained from American Type Culture Collection (Manassas, VA, USA). Cells were maintained in Dulbecco’s modified Eagle’s medium (DMEM; Gibco, New York, USA) supplemented with 10% fetal bovine serum (FBS; Atlanta Biologicals, Lawrenceville, GA, USA) at 37°C under 5% CO_2_. SNE and STE (kindly provided by Dr. Stacy Schultz-Cherry, St. Jude Children’s Research Hospital, Memphis, TN, USA) were cultured at 37°C with 5% CO_2_ in DMEM/F12 (Thermo Fisher Scientific, Asheville, NC, USA) supplemented with fetal bovine serum (10%).

### Viruses and viral propagation

Influenza A/swine/Texas/A01104013/2012(H3N2) virus was propagated for one passage on MDCK cells at 37°C with 5% CO_2_ in Opti-MEM I Reduced Serum Medium (Thermo Fisher Scientific, Asheville, NC, USA) supplemented with 1 μg/mL of TPCK-trypsin (Gibco, New York, USA). Influenza A/mallard/Wisconsin/A00751454/2009(H1N1) virus was propagated for one passage in specific pathogen–free (SPF) 10-day-old chicken embryonated eggs (Charles River Laboratories, Inc., Norwich, CT). The viruses were titrated by the 50% tissue culture infective dose (TCID_50_/mL) on MDCK cells, aliquoted, and stored at −80°C before use. The Genbank access numbers for A/swine/Texas/A01104013/2012(H3N2) virus are JX280447 to JX280454. The genomic sequences for A/mallard/Wisconsin/A00751454/2009 (H1N1) have been deposited into Genbank with access numbers MH879773 to MH879780.

### Viral titration

For viral titration, the TCID_50_ was determined on MDCK cells, and the 50% egg culture infective dose (EID_50_) was determined on SPF 10-day-old chicken embryonated eggs (Charles River Laboratories, Inc., Norwich, CT).

### Hemagglutination and hemagglutination inhibition assays

Hemagglutination and hemagglutination inhibition (HI) assays were carried out by using 0.5% turkey erythrocytes as previously described [58].

### Plaque assay purification of individual virions

Viruses were purified by plaque assay on MDCK cell monolayers as described elsewhere [59]. In brief, nasal wash fluids or the supernatants of ground tissues from experiment animals were 10-fold serially diluted from 10^0^ to 10^−6^ with Opti-MEM containing 100 units/mL of penicillin–streptomycin (Gibco, New York, USA) and inoculated onto monolayers of MDCK cells in 6-well plates. Up to 10 single plaques were randomly picked for each sample. Plaques were propagated on MDCK cells for a single passage, and the viruses were aliquoted and stored at −80°C before being used for genomic sequencing and growth kinetics characterization.

### RNA extraction and genomic sequencing

Viral RNA was extracted by using the KingFisher Pure Viral NA Kit (Thermo Fisher Scientific, Asheville, NC, USA) according to the manufacturer’s instructions. Viral cDNA transcript libraries were prepared using the Nextera XT v2 kit and were sequenced by using a MiSeq Reagent Kit v2 with a MiSeq sequencing system (both from Illumina, San Diego, CA, USA) according to the manufacturer’s suggested protocol [60]. The quality of pair-end reads obtained from MiSeq sequencing were checked by FastQC (Babraham Bioinformatics, https://www.bioinformatics.babraham.ac.uk/projects/fastqc/) and trimmed by Trimmomatic version 0.36 (Usadel Lab, http://www.usadellab.org/cms/?page=trimmomatic) using a quality score threshold of 20. After quality trimming was performed, all pair-end reads were aligned to reference genome of corresponding virus by using Bowtie 2 (Johns Hopkins University, http://bowtie-bio.sourceforge.net/bowtie2/index.shtml). Polymorphisms were identified from alignments and subject to a 20% coverage cutoff.

### Growth kinetics

SNE, STE, A549, DF-1, and MDCK cells were seeded in 6-well plates, after the cells were confluent, cells were washed twice with PBS and then infected with a testing virus at a multiplicity of infection of 0.001 TCID_50_/cell. The inoculum was removed after 1 h of incubation at 37°C. The cells were washed with PBS, and then 3 mL of Opti-MEM supplemented with 1 μg/mL of TPCK-trypsin was added. Cultures were incubated at 33°C, 37°C, or 39°C for the duration of the experiment. At 12, 24, 48, and 72 h post-inoculation, virus titers were determined in the supernatants by TCID_50_ on MDCK cells [59, 61, 62].

### Viral purification and quantification of virus particles

Viruses were purified by sucrose gradient centrifugation. The purified viruses were dissolved in PBS and dialyzed against PBS at 4°C overnight. The concentrations of virus particles were determined using sodium dodecyl sulfate–polyacrylamide gel electrophoresis as described elsewhere [63].

### Virus–glycan receptor binding assay and data analyses

Two biotinlyated glycan analogs, 3ʹSLN and 6ʹSLN, were purchased from GlycoTech (Gaithersburg, MD, USA). Binding of viruses (at 5 nM/virus) to the biotinylated glycan analogs was performed as previously described [64] in an Octet RED96 biolayer interferometer equipped with streptavidin biosensor tips (PALL FortéBIO, Menlo Park, CA, USA). The glycan concentrations ranged from 0.007 μg/mL to 1.5 μg/mL. Responses were normalized by the highest value obtained during the experiment, and binding curves were fitted by using the binding-saturation method in GraphPad Prism version 7 (https://www.graphpad.com/scientific-software/prism/). The normalized response curves report the fractional saturation (*f*) of the sensor surface as described in a previous study [65]. RSL_0.5_ (relative sugar loading, *f* = 0.5) was used to quantitate the binding affinity of two selected viruses against two glycan analogs. The higher the RSL_0.5,_ the smaller the binding affinity.

### Feral swine

For animal experiments, a total of 43 feral swine (body weight 16–22 kg) were trapped in a rural area of Starkville, MS, USA, by using corral traps similar to those previously described [21]. Animals were transported to the National Wildlife Research Center, Mississippi Field Station, in Mississippi State, MS, USA, where they were quarantined for 1 week. Before the animals were included in the experiments, we confirmed that these feral swine had not been exposed to brucellosis, pseudorabies and influenza A virus using ELISA as previously described [21] and that all HI assay results were negative for A/swine/Texas/A01104013/2012(H3N2), A/mallard/Wisconsin/A00751454/2009(H1N1), and three endemic human influenza A viruses [i.e., A/California/04/2009(H1N1), A/Switzerland/9715293/2013(H3N2), and A/Hong Kong/4801/2014(H3N2)]. The swine were then housed and fed according to standard protocol, as described elsewhere [21], and used in three animal experiments.

### Animal experiment for evaluating the pathogenesis and transmission of avian H1N1 virus

To ensure that the selected avian H1N1 virus could cause infection in and be transmitted among swine, we evaluated the pathogenesis and transmission of A/mallard/Wisconsin/A00751454/2009(H1N1) virus in the feral swine prior to the animal study. A total of 12 feral swine were randomly assigned to two groups: the treatment group (n = 8) and the control group (n = 4). The treatment group pigs were housed in 4 adjoining pens (2 pigs/pen); the control pigs were housed in 2 adjoining pens (2 pigs/pen); and the treatment pigs and the control pigs were housed in two individual buildings. Prior to virus inoculation and samples collection, pigs were anesthetized using a method described previously [21]. After being anesthetized, the eight pigs in the treatment group were intranasally inoculated with 10^6^ EID_50_ of virus in a volume of 1 mL (0.5 mL/nostril), and the four pigs in the control group were intranasally inoculated with 1 mL of PBS. On 1–10 dpi, nasal wash fluids were collected from both nostrils of all pigs into 3 mL of PBS and then subjected to titration by EID_50_. The body weight and temperature of each pig were measured before samples were taken. At 5 and 7 dpi, respectively, two treatment group pigs and one control pig were euthanized and necropsy was performed according to a previously described procedure [21]. Turbinates, tracheas, and lungs were collected and homologized for viral titration by EID_50_. Serum from each pig was also collected for seroconversion analysis using an HI assay with 0.5% turkey red blood cells, as described previously [58].

### Animal experiment groups for mixed infections with avian H1N1 and swine H3N2 IAVs

A total of 19 pigs were separated into 5 treatment groups (16 pigs in total) and 1 control group (3 pigs in total). Each treatment group (n = 3 or 4 pigs) consisted of one pig intranasally inoculated on day 0 with 10^6^ TCID_50_ of A/swine/Texas/A01104013/2012(H3N2) virus in a volume of 1 mL; one pig inoculated on day 0 with 10^6^ TCID_50_ of A/mallard/Wisconsin/A00751454/2009(H1N1) virus in a volume of 1 mL; and one or two IAV seronegative pigs to serve as a direct contact(s) for the inoculated pigs. For each experimental group, one or two IAV seronegative pigs were introduced into the same pen housing one pig inoculated with swine H3N2 IAV and one pig inoculated with avian H1N1 IAV right after (at the same day) viral inoculation. To avoid potential contaminations from virus inoculation, the inoculation procedure for each inoculated pig was performed outside of the animal’s housing pens on a stainless steel table which was disinfected between each animal. The control group was housed in a separate building and intranasally inoculated with 1 mL of sterile PBS. Nasal wash fluids were collected from all pigs on 0, 3, 5, and 7 dpi and titrated by TCID_50_ on MDCK cells.

All pigs in two of the five treatment groups were euthanized on 3 dpi and 5 dpi, respectively, and pigs in the remaining treatment group were euthanized on 7 dpi. One control pig each was euthanized on 3, 5, and 7 dpi. The following tissues were collected from each euthanized pig: left cranial lung, left caudal lung, left middle lung, right cranial lung, right caudal lung, right middle lung, right accessory, upper trachea, middle trachea, distal trachea, bronchus, soft palate, ethmoid turbinate, rostral turbinate, and middle turbinate. To quantify IAV, we homogenized each tissue to a 10% (w/v) final concentration in PBS containing 100 unit/mL of penicillin–streptomycin and then subjected the solutions to three freeze–thaw cycles prior to performing virus titrations and plaque analyses.

### Animal experiment for evaluating the pathogenesis and transmission of a reassortant with genes from avian H1N1 and swine H3N2 viruses

We used a total of 12 pigs to evaluate the pathogenesis and transmission of a reassortant (plaque #69 [genotype R3]) which resulted from a mixed infection and has PB1 gene from A/mallard/Wisconsin/A00751454/2009(H1N1) virus and other genes from A/swine/Texas/A01104013/2012(H3N2) virus. We randomly assigned the pigs into four groups of experiment pigs (n = 8) and one group of control pigs (n = 4). Each experiment group consisted of 1 inoculated pig, which received 10^6^ TCID_50_ of a nasal isolate of IAV on day 0, and 1 contact pig. Nasal wash fluids were obtained from each pig on 0, 3, 5, 7, 9, 11, and 14 dpi; serum samples were collected from each pig on 0, 7, 14, and 21dpi. On 3, 5, 7, and 9 dpi, one virus-inoculated pig and one control pig were euthanized, and viruses in nasal wash specimens and animal tissues were titrated by TCID_50_ on MDCK cells; serum samples were subjected to seroconversion analyses by HI assays.

### Data analyses

Viral titers are expressed as means ± standard deviations. The one-way ANOVA test was used to determine whether viral growth in each cell line at 33°C or 39°C differed from growth at 37°C at each time point. Differences with a P-value of less than 0.05 were considered statistically significant.

## Supporting information

S1 Fig Virus load in tissues of feral swine infected with avian H1N1 IAV. Treatment feral swine were intranasally inoculated with 10^6^ EID_50_ avian H1N1; control feral swine were intranasally inoculated with 1 mL PBS. On each indicated day, two inoculated and one control pig were euthanized, and tissues of respiratory track were collected for viral titration in specific pathogen–free eggs. Viral titers were expressed as log_10_EID_50_/mL. Dashed line indicates the limit of detection.
(TIF)

S2 Fig **Variations in body temperature (A) and weight (B) for feral swine infected with avian H1N1 IAV.** The treatment feral swine were intranasally inoculated with 10^6^ TCID_50_ of avian H1N1 virus; control feral swine were intranasally inoculated with 1 mL of PBS. The variations in temperature and weight are expressed as mean ± standard deviation.
(TIF)

S3 Fig Replication efficiency of reassortant viruses in SNE, STE, and A549 cells. Cells were infected at an MOI of 0.001 TCID_50_/cell with the indicated parental or reassortant viruses, and infected SNE cells were incubated at 33°C, STE cells at 37°C, or A549 cells at 39°C. The samples were collected at 72 h post-infection in the supernatant of infected cells in the presence of trypsin-TPCK, and the viral titers were determined by TCID_50_ in MDCK cells. Nasal washes and tissue sections of the testing genotypes of reassortant viruses are shown (N, nasal washes; U, upper respiratory track; M, middle respiratory track; L, lower respiratory track). The origin of each segment for indicated viruses are shown at the right of columns; red indicates segments from swine virus; blue indicates segments from avian virus. If there are polymorphisms identified from different plaques belonging to the same genotype, multiple plaques were selected and marked by star. Data shown represent the mean titer +/− standard error (n = 3 cultures).
(TIF)

S4 Fig Growth dynamics of reassortant viruses with mutation Q197R on HA protein. (A) Two sets of reassortant viruses with different genotypes were selected. Each set contains one virus with mutation 197Q or 197R on HA protein. (B) Viral growth of each selected virus at 72 hours in swine nasal epithelium (SNE), swine trachea epithelium (STE), and A549 cells at 33°C, 37°C, and 39°C, respectively, was determined by TCID_50_ in MDCK cells.
(TIF)

S5 Fig Transmission of virus plaque #69 (genotype R3) in feral swine. Four animals in the treatment group were intranasally inoculated with 10^6^ TCID_50_ of nasal isolate plaque #69 (genotype R3). Another 4 animals (contacts) were introduced into each pen. Nasal washes and tissue of respiratory track were collected from each animal on each indicated day and subjected to viral titer determination by TCID_50_ on MDCK cells. Infectivity was calculated based on the titration result. (A) Infectivity of nasal isolate plaque #69 (genotype R3) in inoculated pigs. (B) Infectivity of nasal isolate plaque #69 (genotype R3) in contact pigs. (C) Serologic responses in contact pigs. Serum was collected from each animal on each indicated day for determination of homologous HI titers. (A, B) solid line indicates the accumulated positiveness rate. (C) dashed line indicates limit of detection. dpi, days postinoculation.
(TIF)

S1 Text (DOCX)

S1 Table Titration of viral shedding in nasal washes from feral swine infected with avian H1N1 IAV. (DOCX)

S2 Table Serologic responses in feral swine infected with avian H1N1 IAV. (DOCX)

S3 Table Titration of virus loads in nasal washes and various tissues from feral swine in co-infection experiments. (DOCX)

S4 Table High frequency amino acid polymorphisms among swine H3N2 (no reassortment) IAV isolates recovered from nasal washes and tissues of feral swine co-infected with avian H1N1 IAV. (DOCX)

S5 Table Polymorphisms of Q197R on HA protein in human and swine H3N2 IAVs downloaded from public databases. (DOCX)

S6 Table Viral shedding in nasal washes from feral swine infected with the nasal isolate plaque #69 (genotype R3). (DOCX)

S7 Table Virus load in various tissues from feral swine infected with the nasal isolate plaque #69 (genotype R3). (DOCX)

S8 Table Serologic responses in feral swine infected with the nasal isolate plaque #69 (genotype R3). (DOCX)

S9 Table Plaques in each genotype. (DOCX)
