# Supplementary figures and images for: Tissue tropisms opt for transmissible reassortants during avian and swine influenza A virus co-infection in swine
Source: PLoS Pathog. 2018 Dec 3;14(12):e1007417. doi: 10.1371/journal.ppat.1007417 (PMC6292640; doi:10.1371/journal.ppat.1007417)

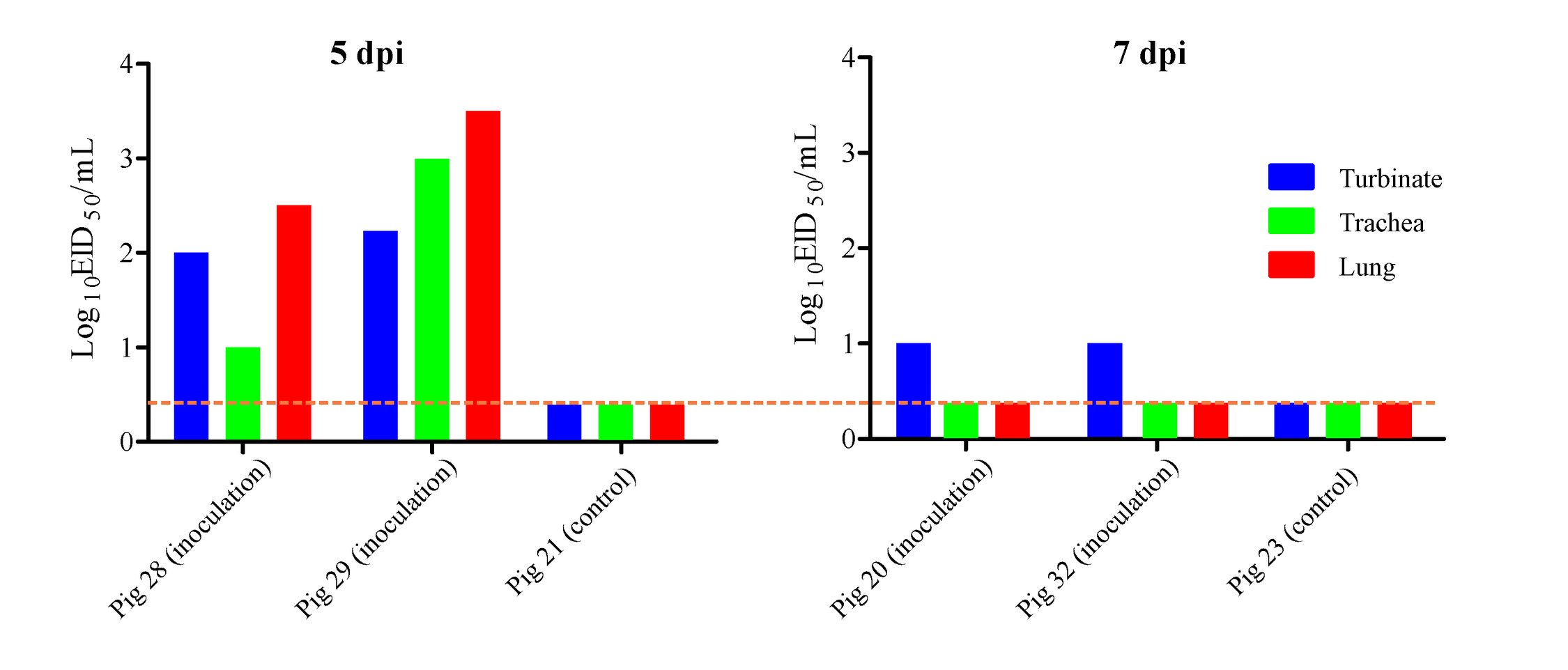

Supplement: S1 Fig — Treatment feral swine were intranasally inoculated with 106 EID50 avian H1N1; control feral swine were intranasally inoculated with 1 mL PBS. On each indicated day, two inoculated and one control pig were euthanized, and tissues of respiratory track were collected for viral titration in specific pathogen–free eggs. Viral titers were expressed as log10EID50/mL. Dashed line indicates the limit of detection. (TIF) [file ppat.1007417.s001.tif]

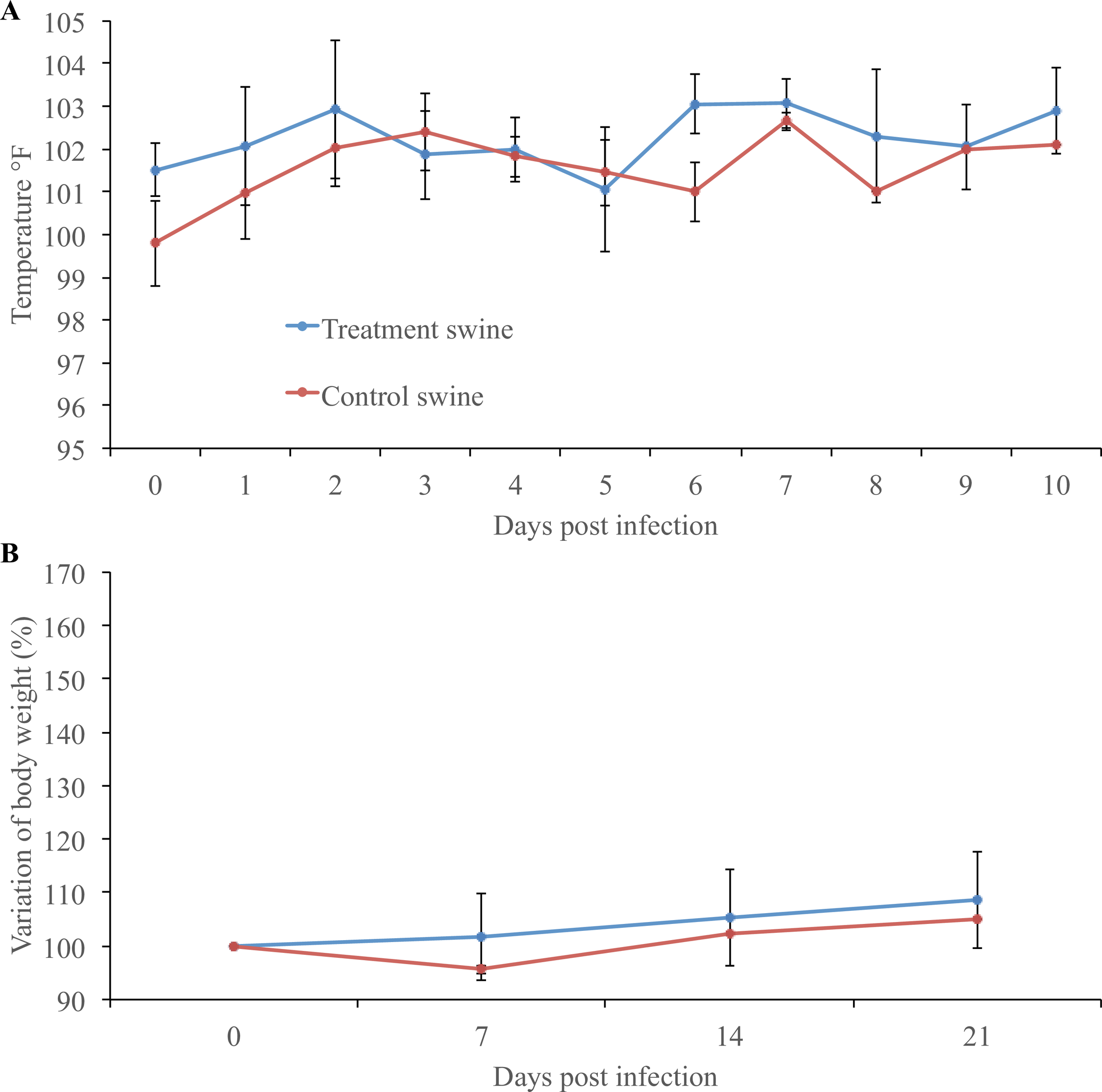

Supplement: S2 Fig — Variations in body temperature (A) and weight (B) for feral swine infected with avian H1N1 IAV. The treatment feral swine were intranasally inoculated with 106 TCID50 of avian H1N1 virus; control feral swine were intranasally inoculated with 1 mL of PBS. The variations in temperature and weight are expressed as mean ± standard deviation. (TIF) [file ppat.1007417.s002.tif]

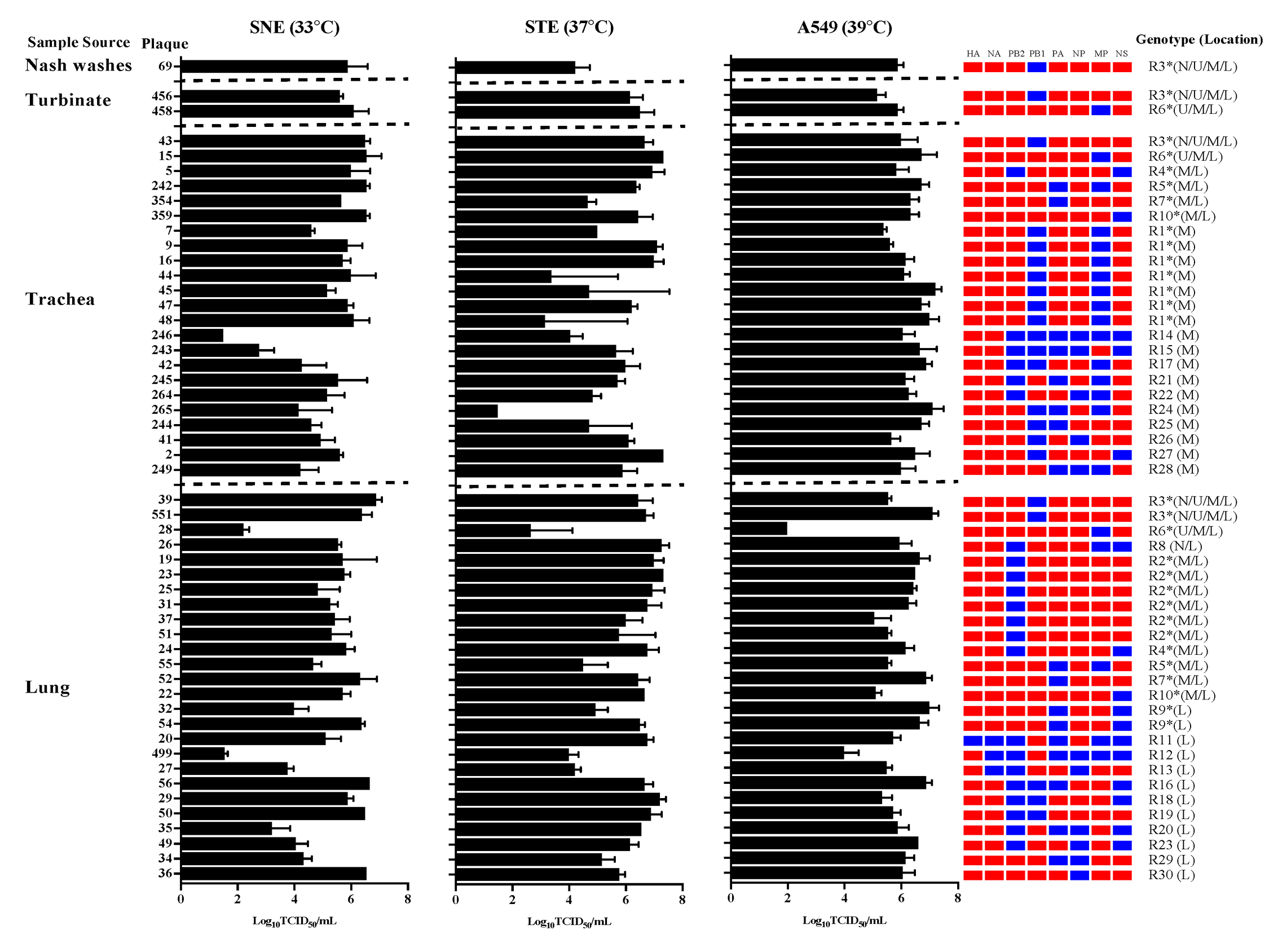

Supplement: S3 Fig — Cells were infected at an MOI of 0.001 TCID50/cell with the indicated parental or reassortant viruses, and infected SNE cells were incubated at 33°C, STE cells at 37°C, or A549 cells at 39°C. The samples were collected at 72 h post-infection in the supernatant of infected cells in the presence of trypsin-TPCK, and the viral titers were determined by TCID50 in MDCK cells. Nasal washes and tissue sections of the testing genotypes of reassortant viruses are shown (N, nasal washes; U, upper respiratory track; M, middle respiratory track; L, lower respiratory track). The origin of each segment for indicated viruses are shown at the right of columns; red indicates segments from swine virus; blue indicates segments from avian virus. If there are polymorphisms identified from different plaques belonging to the same genotype, multiple plaques were selected and marked by star. Data shown represent the mean titer +/− standard error (n = 3 cultures). (TIF) [file ppat.1007417.s003.tif]

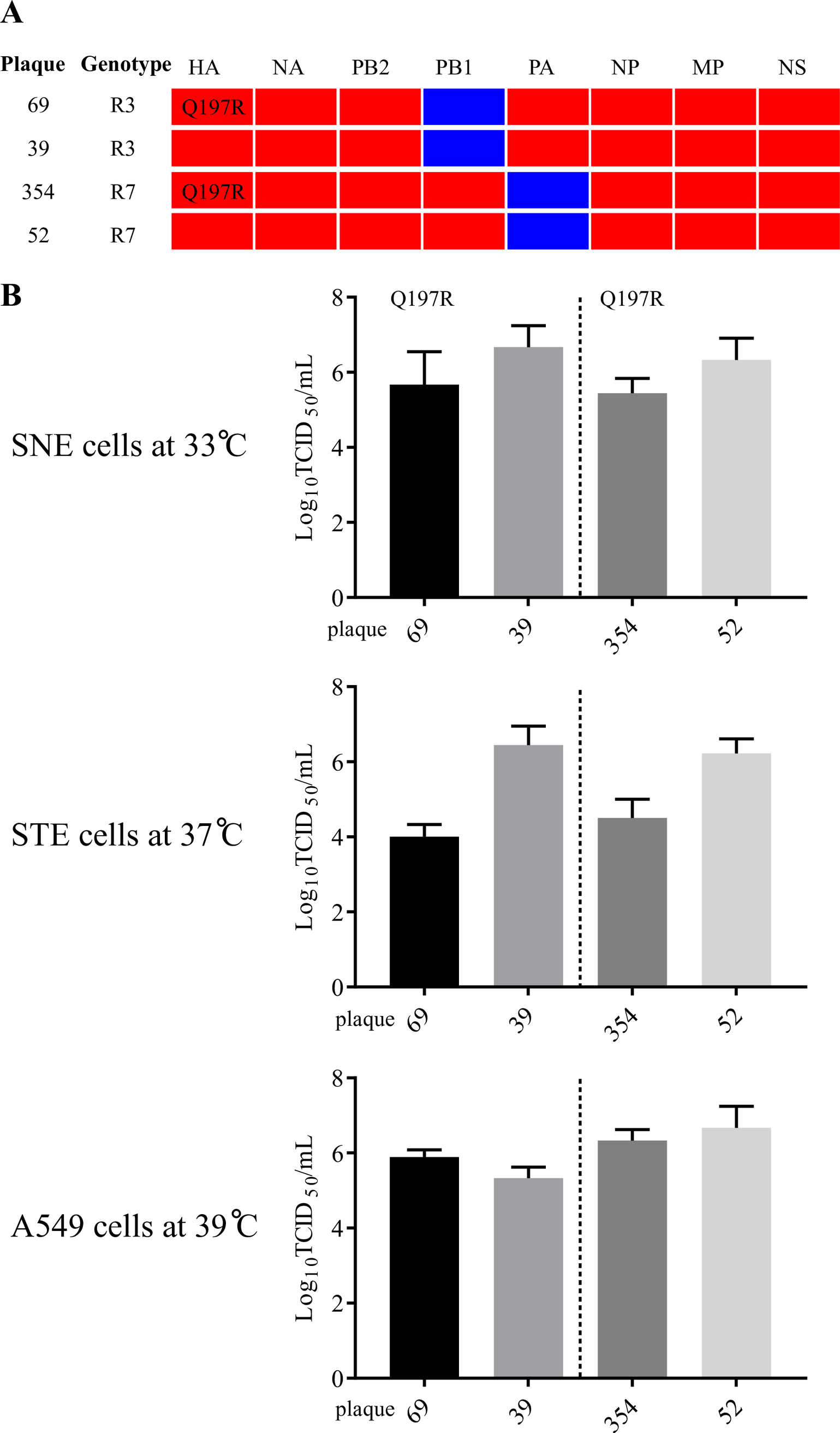

Supplement: S4 Fig — (A) Two sets of reassortant viruses with different genotypes were selected. Each set contains one virus with mutation 197Q or 197R on HA protein. (B) Viral growth of each selected virus at 72 hours in swine nasal epithelium (SNE), swine trachea epithelium (STE), and A549 cells at 33°C, 37°C, and 39°C, respectively, was determined by TCID50 in MDCK cells. (TIF) [file ppat.1007417.s004.tif]

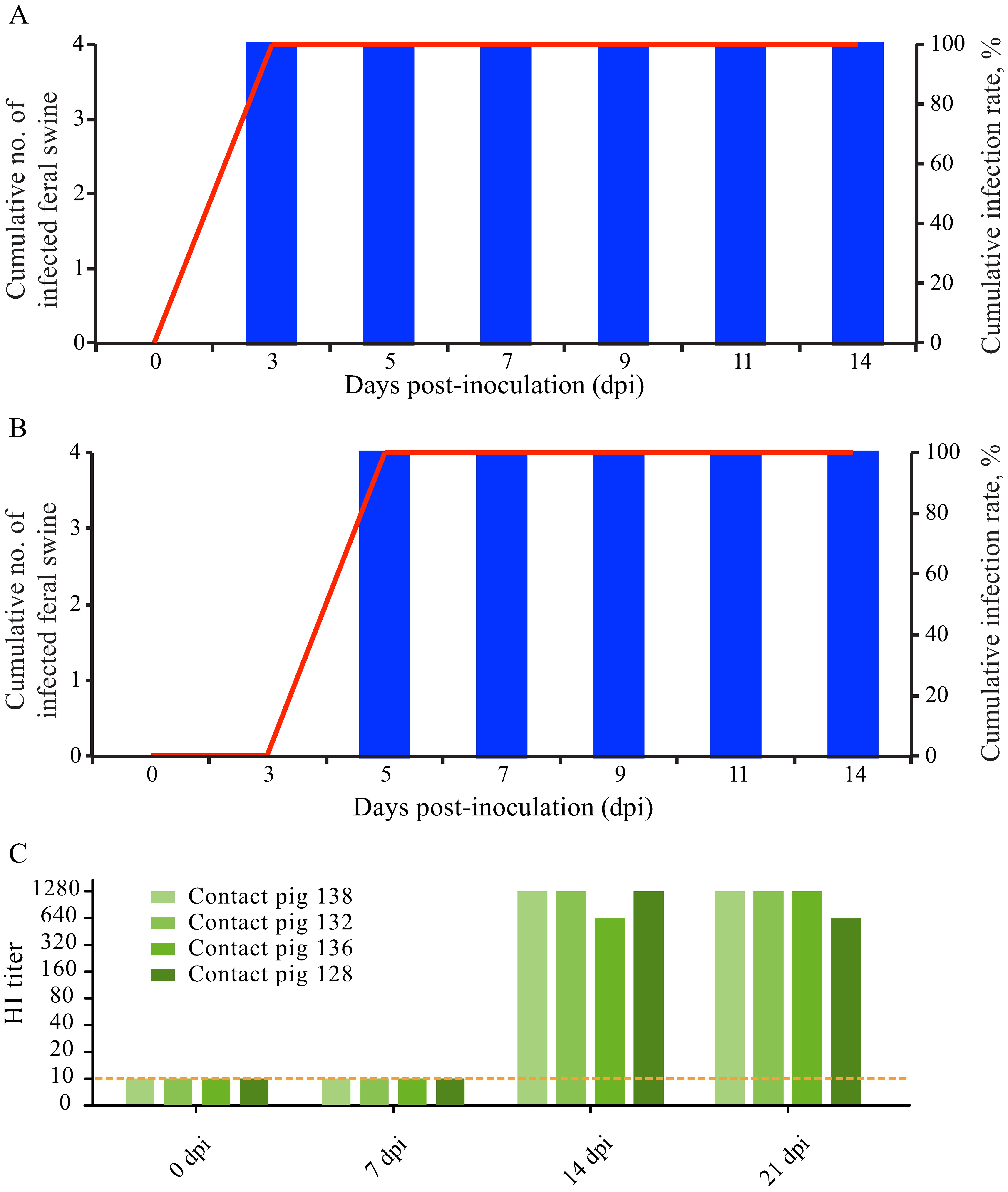

Supplement: S5 Fig — Four animals in the treatment group were intranasally inoculated with 106 TCID50 of nasal isolate plaque #69 (genotype R3). Another 4 animals (contacts) were introduced into each pen. Nasal washes and tissue of respiratory track were collected from each animal on each indicated day and subjected to viral titer determination by TCID50 on MDCK cells. Infectivity was calculated based on the titration result. (A) Infectivity of nasal isolate plaque #69 (genotype R3) in inoculated pigs. (B) Infectivity of nasal isolate plaque #69 (genotype R3) in contact pigs. (C) Serologic responses in contact pigs. Serum was collected from each animal on each indicated day for determination of homologous HI titers. (A, B) solid line indicates the accumulated positiveness rate. (C) dashed line indicates limit of detection. dpi, days postinoculation. (TIF) [file ppat.1007417.s005.tif]
