# Supplementary material for: Tissue tropisms opt for transmissible reassortants during avian and swine influenza A virus co-infection in swine
Source: PLoS Pathog. 2018 Dec 3;14(12):e1007417. doi: 10.1371/journal.ppat.1007417 (PMC6292640; doi:10.1371/journal.ppat.1007417)
Supplement: S1 Text — (DOCX) [file ppat.1007417.s006.docx]

***Supporting Information***

**Tissue tropisms opt for transmissible reassortants during avian and swine influenza A virus co-infection in swine**

**Short title: Reassortment of avian and swine influenza A viruses in swine**

Xiaojian Zhang^1^†, Hailiang Sun^1,2^†, Fred L. Cunningham^3^†, Lei Li^1^†, Katie Hanson-Dorr ^3^, Matthew W. Hopken^4,5^, Jim Cooley^6^, Li-Ping Long^1^, John A. Baroch^4^, Tao Li^7^, Brandon S. Schmit^4^, Xiaoxu Lin^7^, Alicia K. Olivier ^6^, Richard G. Jarman^7^, Thomas J. DeLiberto^4^*, and Xiu-Feng Wan^1^*

**Affiliations:**

^1^Department of Basic Sciences, College of Veterinary Medicine, Mississippi State University, Starkville, Mississippi State, Mississippi, United States;

^2^College of Veterinary Medicine, South China Agricultural University, Guangzhou, Guangdong, China;

^3^Mississippi Field Station, National Wildlife Research Center, Wildlife Services, Animal and Plant Health Inspection Service, US Department of Agriculture, Starkville, Mississippi State, Mississippi, United States;

^4^National Wildlife Research Center, Wildlife Services, Animal and Plant Health Inspection Service, United States Department of Agriculture, Fort Collins, Colorado, United States;

^5^Department of Microbiology, Immunology, and Pathology, Colorado State University, Fort Collins, Colorado State, Colorado, United States;

^6^Department of Pathobiology and Population Medicine, College of Veterinary Medicine, Mississippi State University, Starkville, Mississippi State, Mississippi, United States;

^7^Viral Diseases Branch, Walter Reed Army Institute of Research, Silver Spring, Maryland, United States.

*Correspondence: Dr. Thomas DeLiberto by Thomas.J.DeLiberto@aphis.usda.gov and Dr. Xiu-Feng Wan by [wan@cvm.msstate.edu](mailto:wan@cvm.msstate.edu)

†These authors contributed equally to this work.

**Supplementary Information**

**Infectivity of influenza A subtype H1N1 virus in swine**. Nasal washes from three of eight treatment pigs had titers ranging from 1.00 to 3.00 log_10_[50% egg infective doses (EID_50_)/mL] during postinfection days (dpi) 1–3. Virus loads were detected in at least one of the tissues (turbinate, trachea, or lung) from all four treatment pigs euthanized at 5 dpi and in the turbinate of the other four pigs euthanized at 7 dpi; titers ranged from 1.00 to 3.5 log_10_EID_50_/mL (S1 Fig). No pathogenic changes were observed in the lung and trachea tissues from infected pigs compared with those from control pigs (data not shown). Serologic responses showed that two of four remaining treatment pigs seroconverted (S2 Table); virus titers were detected in nasal washes of only one of the two pigs that seroconverted.

No virus was detected in nasal washes from any of the four sentinel pigs, which were housed in the same room as treatment pigs in adjoining pens (S1 Table). Our data also showed that this avian influenza A H1N1 virus was not transmitted to sentinel pigs housed in the same building.

Neither fever nor significant body weight changes were observed in the treatment pigs (n = 8), which were inoculated with influenza A/mallard/Wisconsin/A00751454/2009(H1N1) virus, or the sentinel pigs (n = 4), which were inoculated with sterile PBS and housed in the same room (S2 Fig). No virus was recovered from fecal swab samples collected from treatment or sentinel pigs (data not shown).

**Temporal and spatial dynamics of genetic reassortments during co-infection**. In total, 571 plaques were generated. The temporal and spatial distributions of plaques were similar to those of the influenza A virus–positive samples (Fig 2D). Of these 571 plaques, 157 were recovered from the samples collected at 3 dpi, 264 at 5 dpi, and 150 at 7 dpi. At 3 dpi, 142 of 157 plaques were from the two pigs inoculated with swine influenza subtype H3N2 virus; these plaques were widely distributed in nasal washes (n = 45) and the upper (n = 48), middle (n = 56), and lower (n = 8) respiratory track tissues. At 5 dpi, plaques were detected in nasal washes (n = 29) and in the upper (n = 43), middle (n = 48), and lower (n = 28) respiratory track tissues of both pigs inoculated with swine H3N2 virus; however, in pigs inoculated with avian H1N1 virus and in contact pigs, plaques were mainly detected in nasal washes (n = 19 and 28, respectively) and upper respiratory tracks (n = 21 and 34, respectively). At 7 dpi, plaques were detected in nasal washes (n = 29) from the pigs inoculated with swine H3N2 virus; in nasal washes (n = 20) and upper respiratory tracks (n = 5) of the pigs inoculated with avian H1N1 virus; and in nasal washes (n = 18) and upper (n = 37), middle (n = 33), and lower (n = 37) respiratory track tissues of the contact pigs.

**Adapted mutations in viruses recovered from co-infections**. Further sequence analysis of isolates showed that the polymorphism rates for HA and PB2 genes are significantly higher than those for other genes. Among 571 virus samples, 277 polymorphisms were detected in the HA, 159 in PB2, 29 in PB1, 48 in PA, 22 in NP, 23 in NA, 20 in MP, and 15 in NS. Furthermore, H1 and H3 influenza viruses have different mutations in HA protein: mutation Q197R is specific for H3 isolates, whereas mutation D418N is specific for H1 isolates. We detected mutation T76N (n = 72 isolates) and V338F (n = 59 isolates) in the PB2 protein. Of interest, in swine H3N2 virus (no reassortment) isolates, mutation Q197R in HA protein exhibits obvious tissue-dependent distribution: more mutations were detected in nasal washes and in the upper respiratory track than in the middle and lower respiratory tracks of feral swine (S4 Table). We further compared the viral growth abilities of plaque viruses with mutation Q197R on HA protein with those without the mutation under three different conditions (S4 Fig). Results showed that, at 72 hours post-inoculation, peak virus titers for plaque viruses with the Q197R mutation on HA protein were lower than those for viruses without this mutation.

Additional analyses on the data from public databases showed that 93.11% of human H3N2 influenza A viruses (IAVs) and 97.95% of swine H3N2 IAVs possess glutamine at residue 197 on HA protein, but just 4.86% of human H3N2 IAVs and 0.61% of swine H3N2 IAVs possess arginine at the same site on HA protein (S5 Table).

**Replication kinetics of reassortant viruses *in vitro***. Fig 4 shows the replication kinetics of eight representative reassortants (1 from nasal wash specimens [R3], 2 from trachea; and 5 from lung) and parent wild type H1N1 and H3N2 viruses. All eight testing reassortants had HA gene from swine H3N2 virus, but at least one of the other seven genes (NA or six internal genes) were from avian H1N1 virus. The analyses were performed at 33°C, 37°C, and 39°C in five cell types: SNE, STE, A549, avian DF-1 (chicken embryo fibroblast), and MDCK cells (S3 Fig). The wild-type swine H3N2 virus replicated efficiently under all testing conditions (5 cell types tested at each of the 3 temperatures) except the setting with DF-1 cells at 39°C. In contrast, wild-type avian H1N1 virus did not replicate well at 33°C regardless of cell types, and did not replicate at all on STE cells at 33°C. Compared with the two wild-type viruses, the 8 testing reassortants had large variations in replication kinetics under the testing conditions. The reassortants R3 (identified in nasal wash fluids and upper, middle, and lower respiratory tracts), R7 (identified in middle and lower respiratory tracts), and R30 (identified in lower respiratory tracts) replicated well in SNE at 33°C and in A549 at 39°C (titers >5.00 log_10_TCID_50_/mL) (S3 Fig). These three reassortants (R3, R7, and R30) had an intermediate replication ability in STE cells at 37°C.
