# Supplementary material for: Tissue tropisms opt for transmissible reassortants during avian and swine influenza A virus co-infection in swine
Source: PLoS Pathog. 2018 Dec 3;14(12):e1007417. doi: 10.1371/journal.ppat.1007417 (PMC6292640; doi:10.1371/journal.ppat.1007417)
Supplement: S1 Table — (DOCX) [file ppat.1007417.s007.docx]

**S1 Table.** **Titration of viral shedding in nasal washes from feral swine infected with avian H1N1 IAV.**

| Group, swine no. | Viral titer (Log_10_EID_50_/mL)^a^ | | | | | | | | | |
| --- | --- | --- | --- | --- | --- | --- | --- | --- | --- | --- |
|  | 1 dpi | 2 dpi | 3 dpi | 4 dpi | 5 dpi | 6 dpi | 7 dpi | 8 dpi | 9 dpi | 10 dpi |
| Infection |  |  |  |  |  |  |  |  |  |  |
| 28 | 2.333 | 3.0 | 2.5 | ND | ND | - | - | - | - | - |
| 29 | ND | ND | ND | ND | ND | - | - | - | - | - |
| 20 | ND | ND | ND | ND | ND | ND | ND | - | - | - |
| 32 | ND | ND | 1.0 | ND | ND | ND | ND | - | - | - |
| 14 | ND | 1.0 | ND | ND | ND | ND | ND | ND | ND | ND |
| 17 | ND | ND | ND | ND | ND | ND | ND | ND | ND | ND |
| 27 | ND | ND | ND | ND | ND | ND | ND | ND | ND | ND |
| 30 | ND | ND | ND | ND | ND | ND | ND | ND | ND | ND |
| Sentinel |  |  |  |  |  |  |  |  |  |  |
| 21 | ND | ND | ND | ND | ND | - | - | - | - | - |
| 23 | ND | ND | ND | ND | ND | ND | ND | - | - | - |
| 25 | ND | ND | ND | ND | ND | ND | ND | ND | ND | ND |
| 26 | ND | ND | ND | ND | ND | - | - | - | - | - |

^a^ND, Viral titers not detectable; -, samples not available.
