# Supplementary material for: Tissue tropisms opt for transmissible reassortants during avian and swine influenza A virus co-infection in swine
Source: PLoS Pathog. 2018 Dec 3;14(12):e1007417. doi: 10.1371/journal.ppat.1007417 (PMC6292640; doi:10.1371/journal.ppat.1007417)
Supplement: S2 Table — (DOCX) [file ppat.1007417.s008.docx]

**S2 Table. Serologic responses in feral swine infected with avian H1N1 IAV.**

| Group, swine no. | HI titer^a^ | | | | |
| --- | --- | --- | --- | --- | --- |
|  | 0 dpi | 7 dpi | 10 dpi | 14 dpi | 21 dpi |
| Infection |  |  |  |  |  |
| 28 | ＜10 | - | - | - | - |
| 29 | ＜10 | - | - | - | - |
| 20 | ＜10 | ＜10 | - | - | - |
| 32 | ＜10 | ＜10 | - | - | - |
| 14 | ＜10 | ＜10 | ＜10 | 40 | 40 |
| 17 | ＜10 | ＜10 | ＜10 | 160 | 40 |
| 27 | ＜10 | ＜10 | ＜10 | ＜10 | ＜10 |
| 30 | ＜10 | ＜10 | ＜10 | ＜10 | ＜10 |
| Sentinel |  |  |  |  |  |
| 21 | ＜10 | - | - | - | - |
| 23 | ＜10 | ＜10 | - | - | - |
| 25 | ＜10 | ＜10 | ＜10 | ＜10 | ＜10 |
| 26 | ＜10 | - | - | - | - |

^a^-, samples not available.
