# Supplementary material for: Tissue tropisms opt for transmissible reassortants during avian and swine influenza A virus co-infection in swine
Source: PLoS Pathog. 2018 Dec 3;14(12):e1007417. doi: 10.1371/journal.ppat.1007417 (PMC6292640; doi:10.1371/journal.ppat.1007417)
Supplement: S3 Table — (DOCX) [file ppat.1007417.s009.docx]

**S3 Table. Titration of virus loads in nasal washes and various tissues from feral swine in co-infection experiments.**

| Euthanasia date, dpi | Pen no./letter | Experimental Group | Pig ID | Viral titer (Log_10_TCID_50_/mL)^a^ | | | | | | | | | | | | | | | | |
| --- | --- | --- | --- | --- | --- | --- | --- | --- | --- | --- | --- | --- | --- | --- | --- | --- | --- | --- | --- | --- |
|  |  |  |  | Nasal wash, dpi | | | | LCR | LCD | RCR | RMD | RCD | RA | TR-U | TR-M | TR-D | BR | RT | MT | ET |
|  |  |  |  | 0 | 3 | 5 | 7 |  |  |  |  |  |  |  |  |  |  |  |  |  |
| 3 | 2 | H1N1 | 62 | ND^a^ | ND | - | - | ND | ND | ND | ND | ND | ND | 0.699 | ND | ND | ND | ND | ND | ND |
| 3 | 2 | H3N2 | 63 | ND | 4.468 | - | - | ND | ND | ND | ND | 3.866 | ND | 4.199 | 4.199 | 4.699 | 4.199 | 4.366 | 4.532 | 4.199 |
| 3 | 2 | Contact | 68 | ND | ND | - | - | ND | ND | ND | ND | ND | ND | ND | ND | ND | ND | ND | ND | ND |
| 3 | 4 | H1N1 | 57 | ND | ND | - | - | ND | ND | ND | ND | ND | ND | ND | ND | ND | ND | ND | ND | ND |
| 3 | 4 | H3N2 | 58 | ND | 4.366 | - | - | ND | ND | ND | ND | ND | ND | 4.199 | 4.199 | 3.090 | ND | 4.699 | 4.699 | 3.199 |
| 3 | 4 | Contact | 59 | ND | ND | - | - | ND | ND | ND | ND | ND | ND | ND | ND | ND | ND | ND | ND | ND |
| 3 | A | Control | 60 | ND | ND | - | - | ND | ND | ND | ND | ND | ND | ND | ND | ND | ND | ND | ND | ND |
| 5 | 6 | H1N1 | 76 | ND | 2.468 | 5.199 | - | ND | ND | ND | ND | ND | ND | ND | ND | 0.699 | 3.137 | 2.881 | 5.032 | 0.699 |
| 5 | 6 | H3N2 | 65 | ND | ND | 4.199 | - | ND | ND | 4.366 | 1.699 | ND | ND | 1.699 | 3.299 | 4.199 | 4.199 | 3.199 | 3.699 | 3.199 |
| 5 | 6 | Contact | 66 | ND | 3.699 | 6.199 | - | ND | ND | ND | ND | ND | ND | ND | ND | ND | ND | 3.366 | 5.366 | 3.468 |
| 5 | 8 | H1N1 | 69 | ND | 2.699 | 0.699 | - | 5.930 | 5.366 | 5.199 | 5.366 | 4.199 | 0.699 | 4.199 | 4.699 | 4.199 | 3.221 | 0.699 | ND | 0.699 |
| 5 | 8 | H3N2 | 70 | ND | 6.032 | 4.199 | - | 6.032 | 5.199 | ND | 4.366 | 4.366 | 5.199 | 4.366 | 4.199 | 4.199 | 5.199 | 3.699 | 3.366 | 0.699 |
| 5 | 8 | Contact | 71 | ND | 1.699 | 4.366 | - | 3.699 | ND | 0.699 | 3.199 | 2.985 | 0.699 | 0.699 | 3.032 | 2.828 | 3.480 | 4.366 | 3.866 | 2.828 |
| 5 | A | Control | 61 | ND | ND | ND | - | ND | ND | ND | ND | ND | ND | ND | ND | ND | ND | ND | ND | ND |
| 7 | 10 | Contact | 72 | ND | ND | 0.699 | 4.930 | 3.866 | 3.480 | 4.366 | 3.137 | ND | 4.199 | 4.366 | 4.366 | 4.199 | 4.366 | 2.699 | 2.063 | 3.824 |
| 7 | 10 | H1N1 | 73 | ND | ND | 3.366 | 4.699 | ND | ND | ND | ND | ND | ND | ND | ND | ND | ND | 2.958 | 3.824 | ND |
| 7 | 10 | H3N2 | 74 | ND | 4.366 | 5.366 | 4.032 | ND | ND | ND | ND | ND | ND | ND | ND | ND | ND | ND | ND | ND |
| 7 | 10 | Contact | 75 | ND | ND | 5.366 | 6.699 | ND | 0.699 | ND | 0.699 | ND | ND | ND | ND | 0.699 | 1.699 | 4.199 | 5.366 | 4.032 |
| 7 | B | Control | 67 | ND | ND | ND | ND | ND | ND | ND | ND | ND | ND | ND | ND | ND | ND | ND | ND | ND |

^a^ND, no virus detected; -, samples not available.
