# Supplementary material for: Tissue tropisms opt for transmissible reassortants during avian and swine influenza A virus co-infection in swine
Source: PLoS Pathog. 2018 Dec 3;14(12):e1007417. doi: 10.1371/journal.ppat.1007417 (PMC6292640; doi:10.1371/journal.ppat.1007417)
Supplement: S4 Table — (DOCX) [file ppat.1007417.s010.docx]

**S4 Table. High frequency amino acid polymorphisms among swine H3N2 (no reassortment) IAV isolates recovered from nasal washes and tissues** **of feral swine co-infected with avian H1N1 IAV**

| Location ^a^ | Viral protein, mutation^b^ | | |
| --- | --- | --- | --- |
|  | HA | PB2 PB2 | |
|  | Q197R | T76N | V338F |
| Nasal wash | 91/157 (57.96) | 30/157 (19.11) | 17/157 (10.83) |
| Upper | 82/181 (45.30) | 24/181 (13.26) | 23/181 (12.71) |
| Middle | 34/115 (29.57) | 10/115 (8.70) | 3/115 (2.61) |
| Low | 9/38 (23.68) | 6/38 (15.79) | 2/38 (5.26) |

^a^Upper, upper respiratory tract; Middle, middle respiratory tract; Lower, lower respiratory tract.^b^Data are no. with polymorphism/no. tested (%).
