# Supplementary material for: Tissue tropisms opt for transmissible reassortants during avian and swine influenza A virus co-infection in swine
Source: PLoS Pathog. 2018 Dec 3;14(12):e1007417. doi: 10.1371/journal.ppat.1007417 (PMC6292640; doi:10.1371/journal.ppat.1007417)
Supplement: S5 Table — (DOCX) [file ppat.1007417.s011.docx]

**S5 Table. Polymorphisms of Q197R on HA protein in human and swine H3N2 IAVs downloaded from public databases.**

| H3N2 IAVs | Amino acid at site 197 of HA protein^a^ | |
| --- | --- | --- |
|  | Q | R |
| Human | 28,033/30,106 (93.11) | 1,464/30,106 (4.86) |
| Swine | 2,586/2,640 (97.95) | 16/2,640 (0.61) |

^a^Data are no. with polymorphism/no. tested (%)
