# Supplementary material for: Tissue tropisms opt for transmissible reassortants during avian and swine influenza A virus co-infection in swine
Source: PLoS Pathog. 2018 Dec 3;14(12):e1007417. doi: 10.1371/journal.ppat.1007417 (PMC6292640; doi:10.1371/journal.ppat.1007417)
Supplement: S6 Table — (DOCX) [file ppat.1007417.s012.docx]

**S6 Table.** **Viral shedding in nasal washes from feral swine infected with the nasal isolate plaque #69 (genotype R3).**

| Pen no./letter | Pig ID | Group | Viral titer (Log_10_TCID_50_/mL) | | | | | |
| --- | --- | --- | --- | --- | --- | --- | --- | --- |
|  |  |  | 3 dpi | 5 dpi | 7 dpi | 9 dpi | 11 dpi | 14 dpi |
| 6 | 129 | Inoculated | 4.67 | - | - | - | - | - |
| 6 | 138 | Contact | ND ^a^ | 6.00 | 6.00 | 3.50 | ND | ND |
| 8 | 133 | Inoculated | 2.67 | 4.00 | - | - | - | - |
| 8 | 132 | Contact | ND | 5.33 | 2.50 | ND | ND | ND |
| 10 | 137 | Inoculated | 3.50 | 6.00 | ND | - | - | - |
| 10 | 136 | Contact | ND | 5.50 | 5.00 | ND | ND | ND |
| 4 | 127 | Inoculated | 2.67 | 4.50 | ND | ND | - | - |
| 4 | 128 | Contact | ND | 6.00 | 4.67 | ND | ND | ND |
| A | 131 | Control | ND | - | - | - | - | - |
| B | 134 | Control | ND | ND | - | - | - | - |
| A | 130 | Control | ND | ND | ND | - | - | - |
| B | 135 | Control | ND | ND | ND | ND | - | - |

^a^ND, viral titers not detectable; -, samples not available.
