# Supplementary material for: Tissue tropisms opt for transmissible reassortants during avian and swine influenza A virus co-infection in swine
Source: PLoS Pathog. 2018 Dec 3;14(12):e1007417. doi: 10.1371/journal.ppat.1007417 (PMC6292640; doi:10.1371/journal.ppat.1007417)
Supplement: S7 Table — (DOCX) [file ppat.1007417.s013.docx]

**S7 Table.** **Virus load in various tissues from feral swine infected with the nasal isolate plaque #69 (genotype R3).**

| Pig ID^a^ | Group | Day of necropsy | Virus load in tissue (Log_10_TCID_50_/mL) | | | | | | | | | | | | | | |
| --- | --- | --- | --- | --- | --- | --- | --- | --- | --- | --- | --- | --- | --- | --- | --- | --- | --- |
|  |  |  | LCR | LCD | LMD | RCR | RCD | RMD | RA | TR-U | TR-M | TR-D | BR | SP | ET | MT | RT |
| 129 | Inoculated | 3 dpi | ND | ND | ND | ND | ND | ND | ND | ND | ND | ND | ND | ND | ND | ND | 5.00 |
| 131 | Control | 3 dpi | ND | ND | ND | ND | ND | ND | ND | ND | ND | ND | ND | ND | ND | ND | ND |
| 133 | Inoculated | 5 dpi | ND | 4.00 | ND | 4.00 | ND | ND | ND | ND | 3.50 | 3.67 | ND | ND | ND | 3.50 | 5.00 |
| 134 | Control | 5 dpi | ND | ND | ND | ND | ND | ND | ND | ND | ND | ND | ND | ND | ND | ND | ND |
| 137 | Inoculated | 7 dpi | ND | ND | ND | ND | ND | ND | ND | ND | ND | ND | ND | ND | ND | ND | ND |
| 130 | Control | 7 dpi | ND | ND | ND | ND | ND | ND | ND | ND | ND | ND | ND | ND | ND | ND | ND |
| 127 | Inoculated | 9 dpi | ND | ND | ND | ND | ND | ND | ND | ND | ND | ND | ND | ND | ND | ND | ND |
| 135 | Control | 9 dpi | ND | ND | ND | ND | ND | ND | ND | ND | ND | ND | ND | ND | ND | ND | ND |

^a^ID, identification; ND, viral titers are not detectable.
