# Supplementary material for: Tissue tropisms opt for transmissible reassortants during avian and swine influenza A virus co-infection in swine
Source: PLoS Pathog. 2018 Dec 3;14(12):e1007417. doi: 10.1371/journal.ppat.1007417 (PMC6292640; doi:10.1371/journal.ppat.1007417)
Supplement: S8 Table — (DOCX) [file ppat.1007417.s014.docx]

**S8 Table. Serologic responses in feral swine infected with the nasal isolate plaque #69 (genotype R3).**

| Pen no./letter | Pig ID^a^ | Group | HI titer^a^ | | | |
| --- | --- | --- | --- | --- | --- | --- |
|  |  |  | 0 dpi | 7 dpi | 14 dpi | 21 dpi |
| 6 | 129 | Inoculated | <10 | - | - | - |
| 6 | 138 | Contact | <10 | <10 | 1,280 | 1,280 |
| 8 | 133 | Inoculated | <10 | - | - | - |
| 8 | 132 | Contact | <10 | <10 | 1,280 | 1,280 |
| 10 | 137 | Inoculated | <10 | 80 | - | - |
| 10 | 136 | Contact | <10 | <10 | 640 | 1,280 |
| 4 | 127 | Inoculated | <10 | 80 | - | - |
| 4 | 128 | Contact | <10 | <10 | 1,280 | 640 |
| A | 131 | Control | <10 | - | - | - |
| B | 134 | Control | <10 | - | - | - |
| A | 130 | Control | <10 | <10 | - | - |
| B | 135 | Control | <10 | <10 | - | - |

^a^ID, identification; -, samples not available.
