# Supplementary material for: Tissue tropisms opt for transmissible reassortants during avian and swine influenza A virus co-infection in swine
Source: PLoS Pathog. 2018 Dec 3;14(12):e1007417. doi: 10.1371/journal.ppat.1007417 (PMC6292640; doi:10.1371/journal.ppat.1007417)
Supplement: S9 Table — (DOCX) [file ppat.1007417.s015.docx]

**S9 Table. Plaques in each genotype.**

| Genotypes | Plaques | | | | | | | |
| --- | --- | --- | --- | --- | --- | --- | --- | --- |
| R1 | 7 | 9 | 13 | 16 | 44 | 45 | 47 | 48 |
| R2 | 10 | 19 | 23 | 25 | 31 | 37 | 51 |  |
| R3 | 39 | 43 | 69 | 258 | 456 | 551 |  |  |
| R4 | 1 | 5 | 21 | 24 | 38 |  |  |  |
| R5 | 18 | 53 | 55 | 242 |  |  |  |  |
| R6 | 15 | 28 | 248 | 458 |  |  |  |  |
| R7 | 52 | 354 | 357 |  |  |  |  |  |
| R8 | 26 | 74 |  |  |  |  |  |  |
| R9 | 32 | 54 |  |  |  |  |  |  |
| R10 | 22 | 359 |  |  |  |  |  |  |
| R11 | 20 |  |  |  |  |  |  |  |
| R12 | 499 |  |  |  |  |  |  |  |
| R13 | 27 |  |  |  |  |  |  |  |
| R14 | 246 |  |  |  |  |  |  |  |
| R15 | 243 |  |  |  |  |  |  |  |
| R16 | 56 |  |  |  |  |  |  |  |
| R17 | 42 |  |  |  |  |  |  |  |
| R18 | 29 |  |  |  |  |  |  |  |
| R19 | 50 |  |  |  |  |  |  |  |
| R20 | 35 |  |  |  |  |  |  |  |
| R21 | 245 |  |  |  |  |  |  |  |
| R22 | 264 |  |  |  |  |  |  |  |
| R23 | 49 |  |  |  |  |  |  |  |
| R24 | 265 |  |  |  |  |  |  |  |
| R25 | 244 |  |  |  |  |  |  |  |
| R26 | 41 |  |  |  |  |  |  |  |
| R27 | 2 |  |  |  |  |  |  |  |
| R28 | 249 |  |  |  |  |  |  |  |
| R29 | 34 |  |  |  |  |  |  |  |
| R30 | 36 |  |  |  |  |  |  |  |
